# An acquired mechanism of antifungal drug resistance simultaneously enables *Candida albicans* to escape from intrinsic host defenses

**DOI:** 10.1371/journal.ppat.1006655

**Published:** 2017-09-27

**Authors:** Irene A. I. Hampe, Justin Friedman, Mira Edgerton, Joachim Morschhäuser

**Affiliations:** 1 Institute for Molecular Infection Biology, University of Würzburg, Würzburg, Germany; 2 Department of Oral Biology, University at Buffalo, Buffalo, New York, United States of America; Boston Children's Hospital, UNITED STATES

## Abstract

The opportunistic fungal pathogen *Candida albicans* frequently produces genetically altered variants to adapt to environmental changes and new host niches in the course of its life-long association with the human host. Gain-of-function mutations in zinc cluster transcription factors, which result in the constitutive upregulation of their target genes, are a common cause of acquired resistance to the widely used antifungal drug fluconazole, especially during long-term therapy of oropharyngeal candidiasis. In this study, we investigated if *C*. *albicans* also can develop resistance to the antimicrobial peptide histatin 5, which is secreted in the saliva of humans to protect the oral mucosa from pathogenic microbes. As histatin 5 has been shown to be transported out of *C*. *albicans* cells by the Flu1 efflux pump, we screened a library of *C*. *albicans* strains that contain artificially activated forms of all zinc cluster transcription factors of this fungus for increased *FLU1* expression. We found that a hyperactive Mrr1, which confers fluconazole resistance by upregulating the multidrug efflux pump *MDR1* and other genes, also causes *FLU1* overexpression. Similarly to the artificially activated Mrr1, naturally occurring gain-of-function mutations in this transcription factor also caused *FLU1* upregulation and increased histatin 5 resistance. Surprisingly, however, Mrr1-mediated histatin 5 resistance was mainly caused by the upregulation of *MDR1* instead of *FLU1*, revealing a previously unrecognized function of the Mdr1 efflux pump. Fluconazole-resistant clinical *C*. *albicans* isolates with different Mrr1 gain-of-function mutations were less efficiently killed by histatin 5, and this phenotype was reverted when *MRR1* was deleted. Therefore, antimycotic therapy can promote the evolution of strains that, as a consequence of drug resistance mutations, simultaneously have acquired increased resistance against an innate host defense mechanism and are thereby better adapted to certain host niches.

## Author summary

The yeast *Candida albicans* is part of the normal microflora of most healthy persons, but it can also cause symptomatic infections when host defenses are compromised. *C*. *albicans* frequently generates genetically altered variants that are better adapted to changes in its environment during colonization and infection. We investigated if *C*. *albicans* can evolve resistance to histatin 5 (Hst 5), an antimicrobial peptide that is produced in the saliva of humans and protects the oral cavity against this pathogen. We found that activated forms of the transcription factor Mrr1 reduce the susceptibility of *C*. *albicans* to killing by Hst 5, a phenotype that was partially caused by Mrr1-mediated overexpression of the multidrug efflux pump *MDR1*. Gain-of-function (GOF) mutations in Mrr1 are a frequent cause of resistance to the antifungal drug fluconazole, especially during long-term treatment of oropharyngeal candidiasis in AIDS patients, but they may also reduce the fitness of the fungus in the absence of the drug. Fluconazole-resistant clinical *C*. *albicans* isolates containing GOF mutations in Mrr1 displayed enhanced Hst 5 resistance, demonstrating that antimycotic therapy can promote the evolution of strains that simultaneously have acquired increased resistance against an innate host defense mechanism and are thereby better adapted to specific host niches.

## Introduction

The yeast *Candida albicans* is a member of the microbiota of the oral cavity and the gastrointestinal and genitourinary tracts in most healthy persons. When host defenses are compromised, *C*. *albicans* can also cause symptomatic infections, which range from superficial skin or mucosal infections to life-threatening, disseminated infections. During both colonization and infection, *C*. *albicans* must adapt to environmental changes and stressful conditions encountered in its various host niches. To a large extent this is achieved by reversibly regulating gene expression and biochemical activities according to cellular needs [1, 2]. In addition, *C*. *albicans* also produces genetically altered variants that are better adapted than the originally colonizing strain to long-lasting changes in its habitat or a new host niche [3–5]. The generation of such variants is facilitated by the high genomic plasticity of this diploid fungus, which often leads to the amplification or loss of partial or whole chromosomes, especially in response to stress [6]. The increased or decreased copy number of genes that are located on the affected chromosomes of the resulting aneuploid cells may confer a selective advantage under certain adverse conditions [7, 8]. Aneuploidies are unstable, and cells can revert to the normal diploid state by chromosome loss or reduplication in the absence of selection pressure [9]. Genetic variants may also arise by simple point mutations that enable the cells to better tolerate harmful conditions. The acquisition of advantageous mutations is frequently followed by loss of heterozygosity for the mutated allele, which can occur by mitotic recombination or loss of the chromosome containing the wild-type allele [10–18]. These events are promoted in a stressful environment and further enhance the effect of the mutations [19, 20].

A well-documented example of such microevolution within the host is the development of antifungal drug resistance during therapy [21]. Infections by *C*. *albicans* are commonly treated with fluconazole, which inhibits the biosynthesis of ergosterol, the main sterol in fungal cell membranes. Mutations in the drug target enzyme sterol 14α-demethylase, encoded by *ERG11*, result in amino acid exchanges that reduce the affinity of the enzyme for the drug [22]. Similarly, mutations in *FKS1*, encoding β-1,3-glucan synthase, the target of echinocandin drugs, also result in reduced drug binding [23]. Other point mutations that cause increased drug resistance affect transcription factors and result in permanently changed gene expression programs. The transcription factor Upc2 regulates the expression of *ERG11* and other ergosterol biosynthesis genes [24, 25]. Gain-of-function (GOF) mutations in Upc2 that result in hyperactivity of the transcription factor cause constitutive upregulation of its target genes and increased fluconazole resistance [15, 26–28]. Similarly, GOF mutations in the transcription factors Mrr1 and Tac1, which regulate the expression of the multidrug efflux pumps *MDR1* and *CDR1*/*CDR2*, respectively, result in constitutive overexpression of their target genes and are responsible for fluconazole resistance in many clinical *C*. *albicans* isolates [11–13, 16, 29–31].

Mrr1, Tac1, and Upc2 belong to the zinc cluster transcription factor family, which is unique to the fungal kingdom and characterized by a well-conserved DNA-binding motif containing six cysteine residues that coordinate two zinc atoms [32]. *C*. *albicans* possesses 82 predicted zinc cluster transcription factors, which are involved in the regulation of diverse cellular processes, although the functions of many of them have not yet been studied in detail [33, 34]. It is conceivable that GOF mutations like those found in Mrr1, Tac1, and Upc2 may also occur in other members of the family and confer new phenotypes that are advantageous under adverse conditions encountered in some host niches. As many transcription factors are activated in response to specific signals and are often not active under standard growth conditions, deletion of the corresponding genes does not necessarily result in an obvious phenotype when the conditions in which they are required are not known. This was the case for Mrr1 and Tac1, whose ability to confer drug resistance was only uncovered by the identification of GOF mutations in fluconazole-resistant clinical isolates [16, 30]. The availability of hyperactive alleles of zinc cluster transcription factors may therefore reveal their biological function and also predict the potential of *C*. *albicans* to generate variants with novel phenotypes by acquiring GOF mutations in these transcriptional regulators. Since it cannot be generally predicted which mutations would render a wild-type transcription factor hyperactive, we recently established a method for the artificial activation of zinc cluster proteins by C-terminal fusion with the heterologous Gal4 activation domain and generated a library of *C*. *albicans* strains expressing all zinc cluster transcription factors of this fungus in a potentially hyperactive form [34]. Screening of this library showed that one of the artificially activated transcription factors, which was thereafter termed Mrr2, conferred fluconazole resistance by upregulation of the major *C*. *albicans* multidrug efflux pump *CDR1* [34]. Based on these findings, other investigators searched for naturally occurring gain-of-function mutations in the *MRR2* gene in a collection of fluconazole-resistant *C*. *albicans* isolates. Indeed, three epidemiologically related isolates with elevated *CDR1* expression levels contained a mutated *MRR2* allele that caused *CDR1* overexpression and increased fluconazole resistance when introduced into a drug-susceptible strain, demonstrating the clinical relevance of the predicted resistance mechanism [35].

In addition to antifungal drugs, which are introduced by medical treatment, *C*. *albicans* encounters many other harmful molecules within its host, which may be taken up with the diet, generated by other members of the microbiota, or produced by the host as a defense mechanism against invading pathogens. Humans secrete saliva containing different antimicrobial peptides, including histatins, in order to protect the oral mucosa from pathogenic microbes. Histatins have strong antifungal activity, with histatin 5 (Hst 5) exhibiting the most potent fungicidal activity against *C*. *albicans* and other *Candida* species [36]. Hst 5 is an unusual antimicrobial peptide in that it is not membrane-lytic but rather acts intracellularly to cause cell death [36]. *C*. *albicans* possesses several mechanisms to evade killing by Hst 5 and thereby can tolerate the presence of low levels of this antimicrobial peptide. The extracellular glycodomain of the plasma membrane protein Msb2 is shed into the environment and binds Hst 5 as well as other antimicrobial peptides, thereby protecting *C*. *albicans* from their action, and extracellular Hst 5 is also proteolytically degraded by secreted aspartic proteases of the fungus [37–39]. Furthermore, *C*. *albicans* can recover from stresses generated by intracellular Hst 5 by mechanisms that are mediated by MAP kinases [40]. As Hst 5 acts within the cells, preventing its intracellular accumulation is another potential resistance mechanism. It was recently shown that the Flu1 efflux pump transports Hst 5 out of the cells and that mutants lacking *FLU1* are hypersusceptible to killing by Hst 5 [41]. We reasoned that *FLU1* expression, like that of the drug efflux pumps *MDR1*, *CDR1*, and *CDR2*, might also be regulated by a zinc cluster protein and that *C*. *albicans* might acquire Hst 5 resistance by GOF mutations in this transcription factor. We, therefore, set out to identify transcription factors that regulate *FLU1* expression and investigate whether *C*. *albicans* can develop increased Hst 5 resistance by this mechanism.

## Results

### Identification of zinc cluster transcription factors that regulate *FLU1* expression

**Fig 1:**
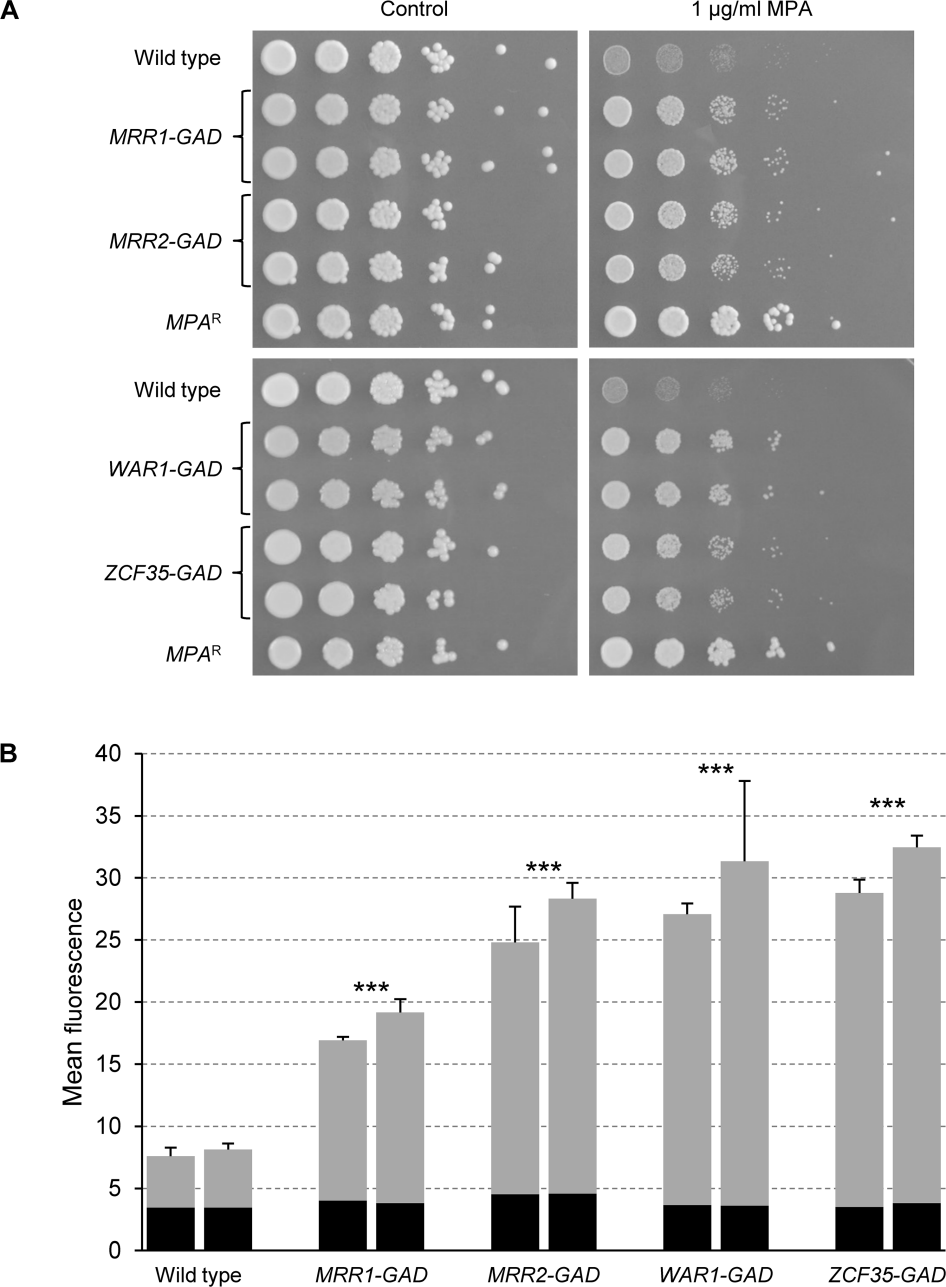
Hyperactive forms of the zinc cluster transcription factors Mrr1, Mrr2, War1, and Zcf35 confer increased MPA resistance and mediate *FLU1* overexpression. (A) Serial dilutions of the strains were spotted onto SD-CSM agar plates without or with 1 μg/ml MPA. The wild-type parent and derivatives expressing the indicated hyperactive transcription factors (two independent transformants in each case) were used in the experiment: SC5314 (Wild type), SCMRR1GAD1A and -B (*MRR1-GAD*), SCZCF34GAD1A and -B (*MRR2-GAD*), SCWAR1GAD1A and -B (*WAR1-GAD*), SCZCF35GAD1A and -B (*ZCF35-GAD*). Strain S2UI1, which carries a mutated *IMH3* allele that confers MPA resistance *(MPA*^R^), was included as an additional control. (B) Strains carrying a P_*FLU1*_*-GFP* reporter fusion and the indicated hyperactive transcription factors were grown to log phase in YPD medium and the mean fluorescence of the cells was determined by flow cytometry. The results obtained with two independently generated reporter strains are shown in each case (means and standard deviations from at least three biological replicates). The background fluorescence of otherwise identical strains without *GFP* is indicated by the black part of each column. *GFP* reporter strains: SCFLU1G2A and -B (Wild type), SCFLU1G2-MRR1GAD1A and -B (*MRR1-GAD*), SCFLU1G2-MRR2GAD1A and -B (*MRR2-GAD*), SCFLU1G2-WAR1GAD1A and -B (*WAR1-GAD*), SCFLU1G2-ZCF35GAD1A and -B (*ZCF35-GAD*). Control strains without *GFP*: SC5314 (Wild type), SCMRR1GAD1A and -B (*MRR1-GAD*), SCZCF34GAD1A and -B (*MRR2-GAD*), SCWAR1GAD1A and -B (*WAR1-GAD*), SCZCF35GAD1A and -B (*ZCF35-GAD*). Significant differences from the wild-type control (background-subtracted values) are marked with asterisks (*** *P* < 0.001; ANOVA).

If *FLU1*, like other known *C*. *albicans* efflux pumps, is regulated by a zinc cluster protein, a hyperactive form of this transcription factor should cause constitutive *FLU1* overexpression and, consequently, increased Hst 5 resistance. *flu1*∆ mutants not only exhibit increased susceptibility to Hst 5 but are also hypersensitive to mycophenolic acid (MPA), a phenotype that is easily recognizable on agar plates containing a suitable concentration of the drug [42]. We therefore screened our library of *C*. *albicans* strains expressing artificially activated forms of all zinc cluster transcription factors for increased MPA resistance. In a preliminary test, the strains were directly transferred from our stock collection in microtiter plates onto agar plates with and without MPA. Candidate strains that grew better than the wild-type control in the presence of the inhibitor were then retested in a more sensitive dilution spot assay. This resulted in the identification of four hyperactive transcription factors (Mrr1, Mrr2, War1, and Zcf35) that caused clearly increased MPA resistance (Fig 1A).

MPA resistance might be brought about by different mechanisms, for example by upregulation of the *IMH3* gene, which encodes inosine monophosphate dehydrogenase, the target enzyme of MPA [43]. To investigate if the increased MPA resistance of our strains was caused by *FLU1* overexpression, we introduced the artificially activated transcription factors into reporter strains containing *GFP* under the control of the *FLU1* promoter. As can be seen in Fig 1B, basal *FLU1* expression levels were detectable with *GFP* as a reporter gene, because the fluorescence of the wild-type reporter strains was well above background fluorescence. Intriguingly, *FLU1* promoter activity was increased by all four hyperactive transcription factors (3- to 6-fold), indicating that Mrr1, Mrr2, War1, and Zcf35 regulate *FLU1* expression and that their activation results in overexpression of this efflux pump.

**Fig 2:**
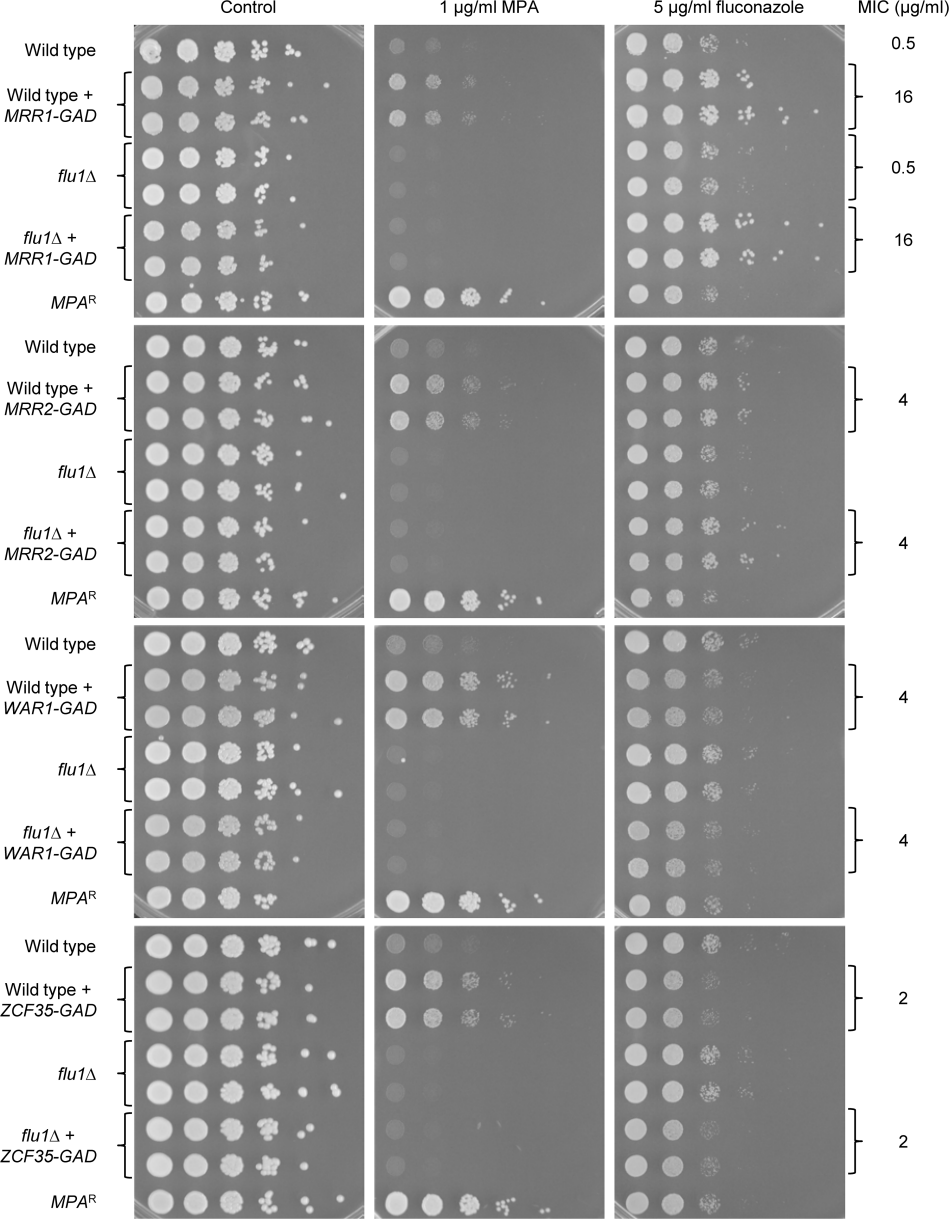
MPA resistance conferred by hyperactive Mrr1, Mrr2, War1, and Zcf35 depends on *FLU1*. Serial dilutions of the wild type, *flu1*∆ mutants, and transformants expressing the indicated hyperactive transcription factors were spotted onto SD-CSM agar plates without or with 1 μg/ml MPA or 5 μg/ml fluconazole and grown for 2 days at 30°C. The MICs of fluconazole, as determined in a broth microdilution assay, are given on the right. The following strains were used in these experiments: SC5314 (Wild type), SCFLU1M4A and -B (*flu1*∆), SCMRR1GAD1A and -B (Wild type + *MRR1-GAD*), SCΔ*flu1*MRR1GAD1A and -B (*flu1*∆ + *MRR1-GAD*), SCZCF34GAD1A and -B (Wild type + *MRR2-GAD*), SCΔ*flu1*MRR2GAD1A and -B (*flu1*∆ + *MRR2-GAD*), SCWAR1GAD1A and -B (Wild type + *WAR1-GAD*), SCΔ*flu1*WAR1GAD1A and -B (*flu1*∆ + *WAR1-GAD*), SCZCF35GAD1A and -B (Wild type + *ZCF35-GAD*), SCΔ*flu1*ZCF35GAD1A and -B (*flu1*∆ + *ZCF35-GAD*). Strain S2UI1, which carries a mutated *IMH3* allele that confers MPA resistance *(MPA*^R^), was included as an additional control.

We next tested if the increased MPA resistance conferred by hyperactive forms of Mrr1, Mrr2, War1, and Zcf35 depended on Flu1. For this purpose, we generated two independent series of *flu1*∆ mutants and complemented strains of the wild-type strain SC5314. In accord with a previous report [42], homozygous *flu1*∆ mutants were hypersusceptible to MPA, and reintroduction of a functional *FLU1* copy into these mutants increased their MPA resistance to the level observed for heterozygous mutants (S1 Fig). The genes encoding the hyperactive transcription factors were then introduced into the homozygous *flu1*∆ mutants and the MPA susceptibilities of the resulting strains compared with those of the corresponding wild-type strains. Fig 2 shows that none of the hyperactive transcription factors caused a noticeable increase in MPA resistance in the absence of Flu1, indicating that this effect on the phenotype of the cells was entirely Flu1-dependent.

Although *FLU1* overexpression in the heterologous host *Saccharomyces cerevisiae* had previously been found to result in elevated fluconazole resistance (hence the name given to the gene), deletion of *FLU1* in a *C*. *albicans* laboratory strain had little effect on fluconazole susceptibility [42]. In line with these findings, we did not observe a detectable increase in fluconazole susceptibility of the *flu1*∆ mutants derived from the wild-type strain SC5314, neither in dilution spot assays on agar plates nor when we determined the minimal inhibitory concentration of fluconazole in a broth microdilution assay (Fig 2). Hyperactive forms of Mrr1, Mrr2, and Zcf35 cause increased fluconazole resistance [34]. For Mrr1 and Mrr2, this was also evident in the dilution spot assays on agar plates containing a defined concentration of fluconazole, whereas the strains with the hyperactive Zcf35 showed even slightly reduced growth on the fluconazole plates, despite the elevated MIC for these strains (Fig 2). The strains containing the hyperactive War1 also showed slightly reduced growth on the fluconazole agar plates, but exhibited increased fluconazole resistance when tested in the MIC assays, a phenotype that was not observed for these strains in our previous study, presumably because a different assay medium was used. The increased fluconazole resistance conferred by the four hyperactive transcription factors was also observed when they were expressed in *flu1*∆ mutants (Fig 2), demonstrating that *FLU1* overexpression did not contribute to the fluconazole-resistant phenotype.

Although *FLU1* mediates Hst 5 resistance, its expression is not induced by Hst 5 [41]. Therefore, we investigated if basal *FLU1* expression levels depend on any of the identified transcription factors. For this purpose, the P_*FLU1*_*-GFP* reporter fusion was introduced into *mrr1*∆, *mrr2*∆, *war1*∆, and *zcf35*∆ mutants. S2 Fig shows that all mutants displayed wild-type *FLU1* promoter activity, indicating that none of these transcription factors is required for basal *FLU1* expression.

### A hyperactive Mrr1 confers increased Hst 5 resistance

**Fig 3:**
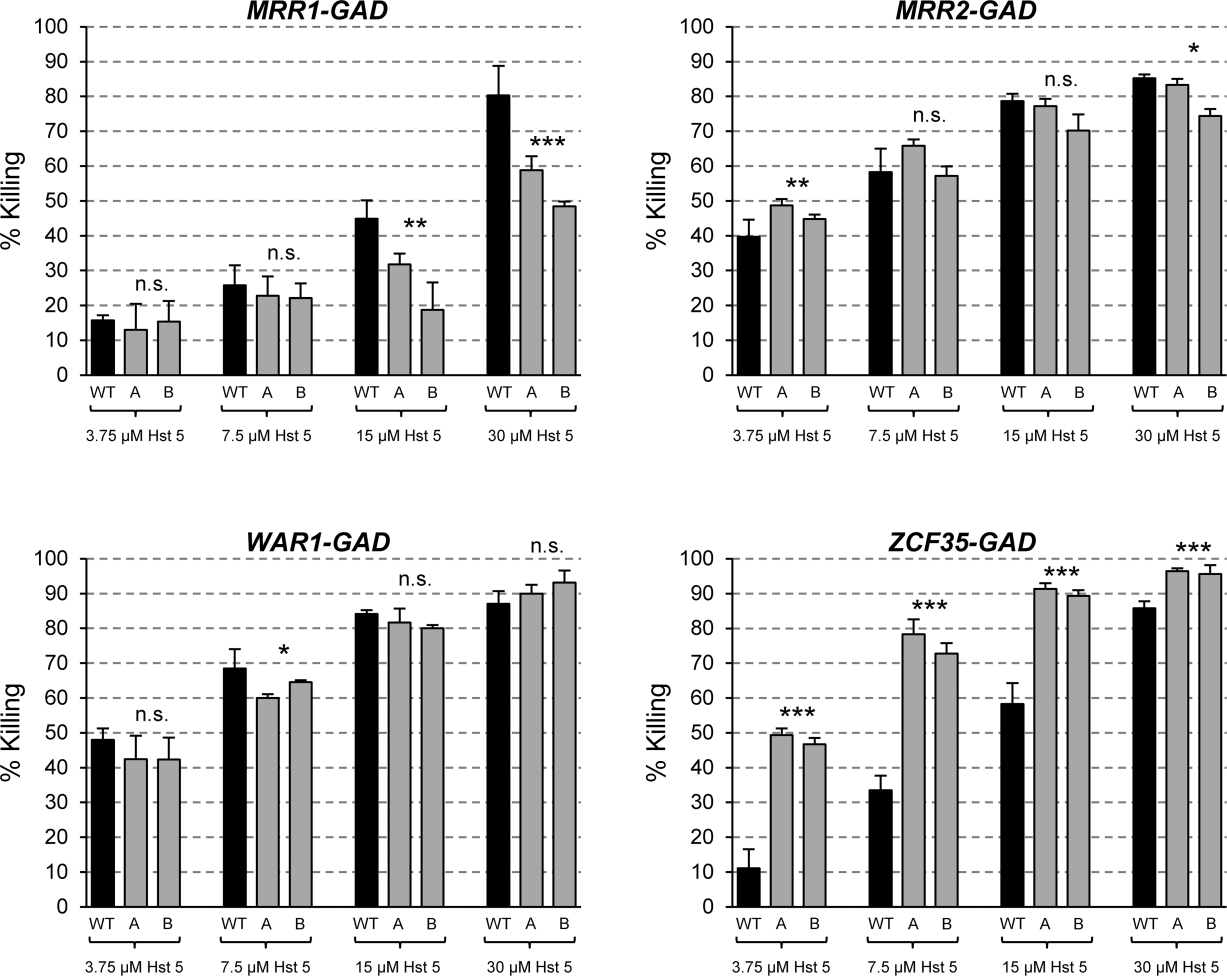
A hyperactive Mrr1 causes increased Hst 5 resistance. The wild-type strain SC5314 (WT, black bars) and derivatives (gray bars) containing artificially activated *MRR1* (SCMRR1GAD1A and -B), *MRR2* (SCZCF34GAD1A and -B), *WAR1* (SCWAR1GAD1A and -B), or *ZCF35* (SCZCF35GAD1A and -B) were exposed to Hst 5 (3.75 μM to 30 μM) using microdilution plate assays, and the percent killing was calculated as described in Materials and Methods. The four transcription factors were tested on separate occasions, and a different batch of Hst 5 was used for *MRR1* and *ZCF35 vs*. *MRR2* and *WAR1*. Significant differences from the wild-type control (t-test) are marked with asterisks (* *P* < 0.05, ** *P* < 0.01, *** *P* < 0.001); n.s., not significant (*P* > 0.05).

As hyperactive forms of Mrr1, Mrr2, War1, and Zcf35 cause *FLU1* upregulation, we reasoned that they should also mediate increased resistance to Hst 5. Therefore, we compared the percent killing of the wild-type parental strain SC5314 and derivatives containing the artificially activated transcription factors after 60 min of incubation in the presence of various Hst 5 concentrations (Fig 3). The hyperactive Mrr1 indeed conferred increased resistance to the antimicrobial peptide; in the presence of 30 μM Hst 5, killing was reduced from 80% for the wild type to *ca*. 54% for strains containing the artificially activated Mrr1. In contrast, strains with the hyperactive Mrr2 and War1 were as efficiently killed by Hst 5 as the wild type, and the strains with the hyperactive Zcf35 showed even enhanced sensitivity. Therefore, despite the increased *FLU1* expression levels, artificially activated Mrr2, War1, and Zcf35 were unable to confer Hst 5 resistance. We speculated that these hyperactive transcription factors have additional effects on the cells that increase their susceptibility to Hst 5 and thereby abrogate any advantage conferred by *FLU1* overexpression. However, subsequent experiments provided a different explanation for this result (see below).

### Naturally occurring GOF mutations in Mrr1 cause *FLU1* overexpression and increased Hst 5 resistance

Our finding that an artificially activated form of Mrr1 causes increased Hst 5 resistance was particularly intriguing, since many fluconazole-resistant clinical *C*. *albicans* isolates contain GOF mutations in Mrr1 [13, 16, 44]. Such mutations are selected under antifungal therapy, because the hyperactive transcription factor mediates overexpression of the multidrug efflux pump *MDR1* and other genes, and thereby confers fluconazole resistance. We investigated if such naturally occurring Mrr1 GOF mutations also cause *FLU1* overexpression and enhanced Hst 5 resistance. Mrr1 GOF mutations have a much stronger effect on *MDR1* expression and fluconazole resistance after loss of heterozygosity for the mutated *MRR1* allele [16, 45], and we reasoned that the same would be true for any effect on *FLU1* expression and Hst 5 resistance. To assess the effect of such mutations in an isogenic background, we replaced both endogenous *MRR1* alleles of strain SC5314 by alleles with GOF mutations that were originally discovered in fluconazole-resistant clinical isolates and resulted in the amino acid exchanges P683S, G997V, G878E, Q350L, N803D, T360I, K335N, and T896I. The clinical isolate 6692 contains different GOF mutations (T360I and K335N) in its two *MRR1* alleles [13]. To reproduce this scenario, we also constructed strains containing a combination of these hyperactive alleles. In addition, strains in which the endogenous *MRR1* alleles were replaced in the same fashion by an unmutated wild-type allele were included in the experiments to make sure that the sequential allele replacement strategy alone did not have a phenotypic effect.

**Fig 4:**
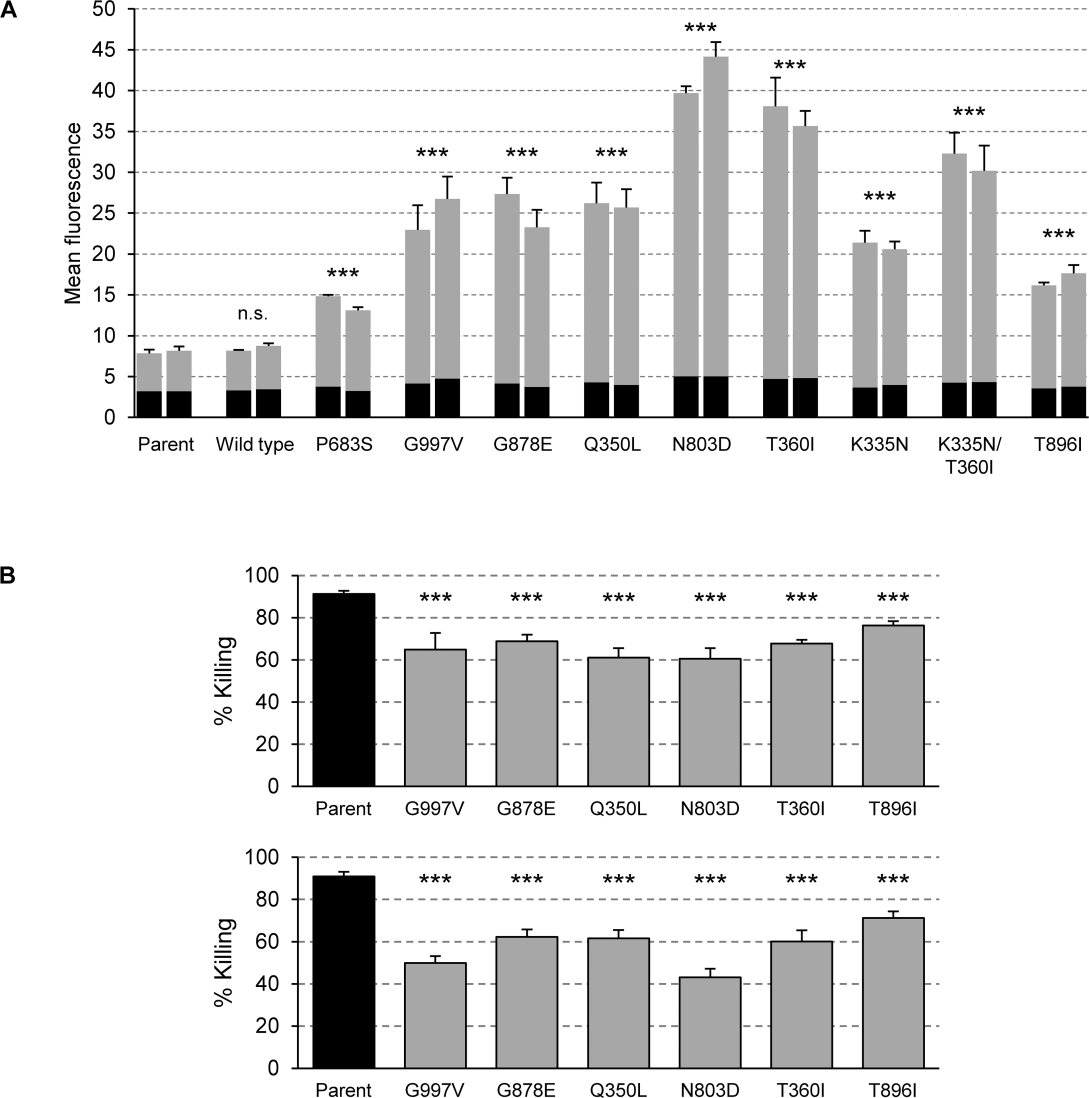
Naturally occuring Mrr1 GOF mutations cause *FLU1* overexpression and increased Hst 5 resistance. (A) Activation of the *FLU1* promoter by different Mrr1 GOF mutations. The two endogenous *MRR1* alleles of the parental strain SC5314 were replaced by mutated alleles encoding the indicated amino acid exchanges or by a nonmutated control allele (wild type). A P_*FLU1*_*-GFP* reporter fusion was integrated into two independently constructed strains in each case. Strains were grown to log phase in YPD medium and the mean fluorescence of the cells was determined by flow cytometry (means and standard deviations from at least three biological replicates). The background fluorescence of the parental strains without *GFP* is indicated by the black part of the columns. *GFP* reporter strains: SCFLU1G2A and -B (Parent), SCMRR1R24FLU1G2A and -B (Wild type), SCMRR1R34FLU1G2A and -B (P683S), SCMRR1R44FLU1G2A and -C (G997V), SCMRR1R54FLU1G2A and -B (G878E), SCMRR1R64FLU1G2A and -B (Q350L), SCMRR1R74FLU1G2A and -B (N803D), SCMRR1R84FLU1G2A and -B (T360I), SCMRR1R94FLU1G2A and -B (K335N), SCMRR1R982FLU1G2A and -B (K335N/T360I), SCMRR1R104FLU1G2A and -B (T896I). Control strains without *GFP*: SC5314 (Parent), SCMRR1R24A and -B (Wild type), SCMRR1R34A and -B (P683S), SCMRR1R44A and -B (G997V), SCMRR1R54A and -B (G878E), SCMRR1R64A and -B (Q350L), SCMRR1R74A and -B (N803D), SCMRR1R84A and -B (T360I), SCMRR1R94A and -B (K335N), SCMRR1R982A and -B (K335N/T360I), SCMRR1R104A and -B (T896I). Significant differences from the parent control (background-subtracted values, ANOVA) are marked with asterisks (*** *P* < 0.001); n.s., not significant (*P* > 0.05). (B) Natural GOF mutations in Mrr1 cause increased Hst 5 resistance. Strains were incubated in the absence or presence of 30 μM Hst 5 and the percent killing was determined as described in Materials and Methods. Two sets of experiments were performed on separate occasions: The top panel shows the comparison of the A-series of mutants with the parental strain SC5314, and the bottom panel shows the comparison of the B-series of mutants with the parental strain SC5314. The following strains were used in these experiments: SC5314 (Parent), SCMRR1R44A and -C (G997V), SCMRR1R54A and -B (G878E), SCMRR1R64A and -B (Q350L), SCMRR1R74A and -B (N803D), SCMRR1R84A and -B (T360I), SCMRR1R104A and -B (T896I). Significant differences from the parent control (ANOVA) are marked with asterisks (*** *P* < 0.001); n.s., not significant (*P* > 0.05).

We first tested the fluconazole susceptibilities of the strains (Table 1). As previously reported [20], replacement of the endogenous *MRR1* alleles of strain SC5314 by a nonmutated *MRR1* copy did not affect drug susceptibility, whereas the P683S mutation caused a 32-fold increase in the MIC of fluconazole (from 0.5 μg/ml to 16 μg/ml). A similar effect was observed for all other GOF mutations, which raised the MIC to 16 or 32 μg/ml. In each case, identical results were obtained for two independently constructed strains. We then introduced the P_*FLU1*_*-GFP* reporter fusion into the strains with the different *MRR1* GOF mutations to determine their effect on *FLU1* expression. Fig 4A shows that all hyperactive *MRR1* alleles caused elevated *FLU1* expression levels, albeit to various degrees. The strongest upregulation (*ca*. 7-fold) was mediated by the N803D and T360I mutations, and the weakest effect (2- to 3-fold upregulation) was observed for the P683S and T896I mutations. The differences were reproducible, because in all cases the two independently constructed reporter strains exhibited similar fluorescence values. These results demonstrated that the overexpression of *FLU1* by a hyperactive Mrr1 is not a peculiar effect of the artificially generated Mrr1-GAD fusion but a general characteristic of naturally occurring activated forms of Mrr1.

**Table 1:** Fluconazole MICs for isogenic strains with different GOF mutations in *MRR1*.

Strain	*MRR1* alleles	MIC Fluconazole (μg/ml)
SC5314	*MRR1*/*MRR1*	0.5
SCMRR1R24A and -B	*MRR1*/*MRR1*	0.5
SCMRR1R34A and -B	*MRR1*^P683S^/*MRR1*^P683S^	16
SCMRR1R44A and -C	*MRR1*^G997V^/*MRR1*^G997V^	16
SCMRR1R54A and -B	*MRR1*^G878E^/*MRR1*^G878E^	32
SCMRR1R64A and -B	*MRR1*^Q350L^/*MRR1*^Q350L^	32
SCMRR1R74A and -B	*MRR1*^N803D^/*MRR1*^N803D^	32
SCMRR1R84A and -B	*MRR1*^T360I^/*MRR1*^T360I^	32
SCMRR1R94A and -B	*MRR1*^K335N^/*MRR1*^K335N^	32
SCMRR1R982A and -B	*MRR1*^K335N^/*MRR1*^T360I^	32
SCMRR1R104A and -B	*MRR1*^T896I^/*MRR1*^T896I^	16

We selected six different Mrr1 GOF mutations (G997V, G878E, Q350L, N803D, T360I, T896I) to investigate if they also resulted in increased Hst 5 resistance. As can be seen in Fig 4B, all strains containing hyperactive *MRR1* alleles were less efficiently killed by Hst 5 than the parental wild-type strain SC5314 (killing decreased from *ca*. 90% to *ca*. 60%), demonstrating that naturally occurring Mrr1 GOF mutations conferred increased Hst 5 resistance. In a further control experiment we confirmed that the strains in which the endogenous *MRR1* alleles were replaced by a nonmutated wild-type copy exhibited wild-type Hst 5 susceptibility, as expected (S3 Fig).

### Mrr1-mediated Hst 5 resistance does not depend on Flu1

**Fig 5:**
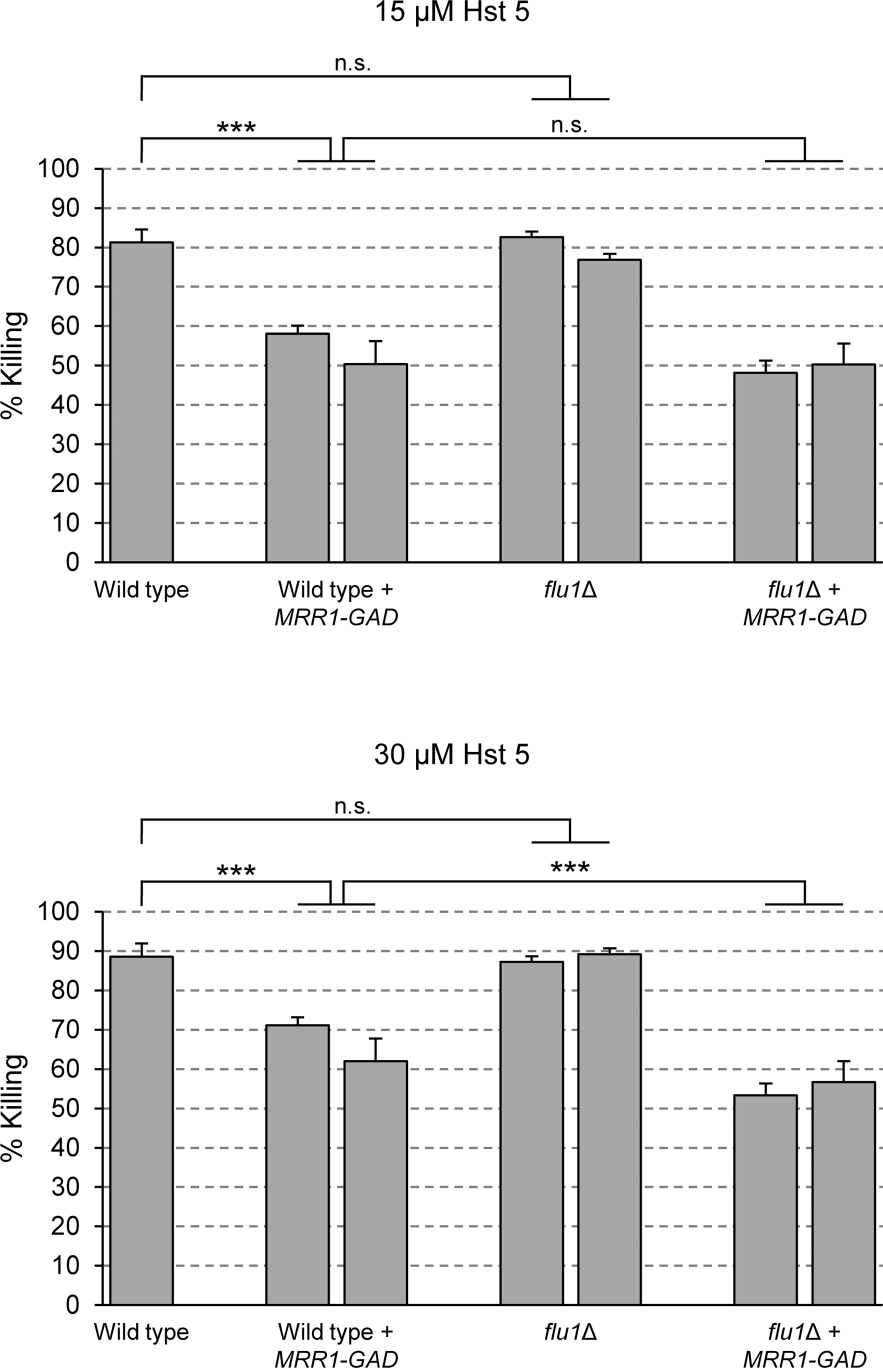
Mrr1-mediated Hst 5 resistance does not depend on Flu1. Wild type and *flu1*∆ mutants as well as derivatives of these strains containing the artificially activated *MRR1* were incubated in the absence or presence of 15 μM or 30 μM Hst 5 and the percent killing was determined as described in Materials and Methods. The following strains were used in this experiment: SC5314 (Wild type), SCMRR1GAD1A and -B (Wild type + *MRR1-GAD*), SCFLU1M4A and -B (*flu1*∆), SCΔ*flu1*MRR1GAD1A and -B (*flu1*∆ + *MRR1-GAD*). Significant differences (ANOVA) are marked with asterisks (*** *P* < 0.001); n.s., not significant (*P* > 0.05).

Despite the fact that hyperactive forms of Mrr2, War1, and Zcf35 caused even stronger *FLU1* upregulation than the artificially activated Mrr1 (see Fig 1B), only the latter enhanced the resistance of the cells to Hst 5 (Fig 3). We therefore investigated if the increased Hst 5 resistance mediated by a hyperactive Mrr1 was indeed caused by *FLU1* overexpression. For this purpose, we compared the percent killing by Hst 5 of wild-type and *flu1*∆ cells with and without the artificially activated *MRR1* allele (Fig 5). Unexpectedly, deletion of *FLU1* in the prototrophic wild-type strain SC5314 did not result in hypersensitivity to Hst 5, in contrast to previous observations with the auxotrophic laboratory strain CAF4-2 [41]. Even more surprisingly, the hyperactive Mrr1 reduced the susceptibility of the cells to killing by Hst 5 both in the presence and absence of *FLU1* with comparable efficiency (at 30 μM Hst 5, the *flu1*∆ mutants were even slightly more resistant in this experiment). Therefore, Mrr1-mediated Hst 5 resistance must be caused by other mechanisms, which might also mask any additional contribution of *FLU1* overexpression in this strain background.

### Overexpression of the multidrug efflux pump *MDR1* contributes to Mrr1-mediated Hst 5 resistance

**Fig 6:**
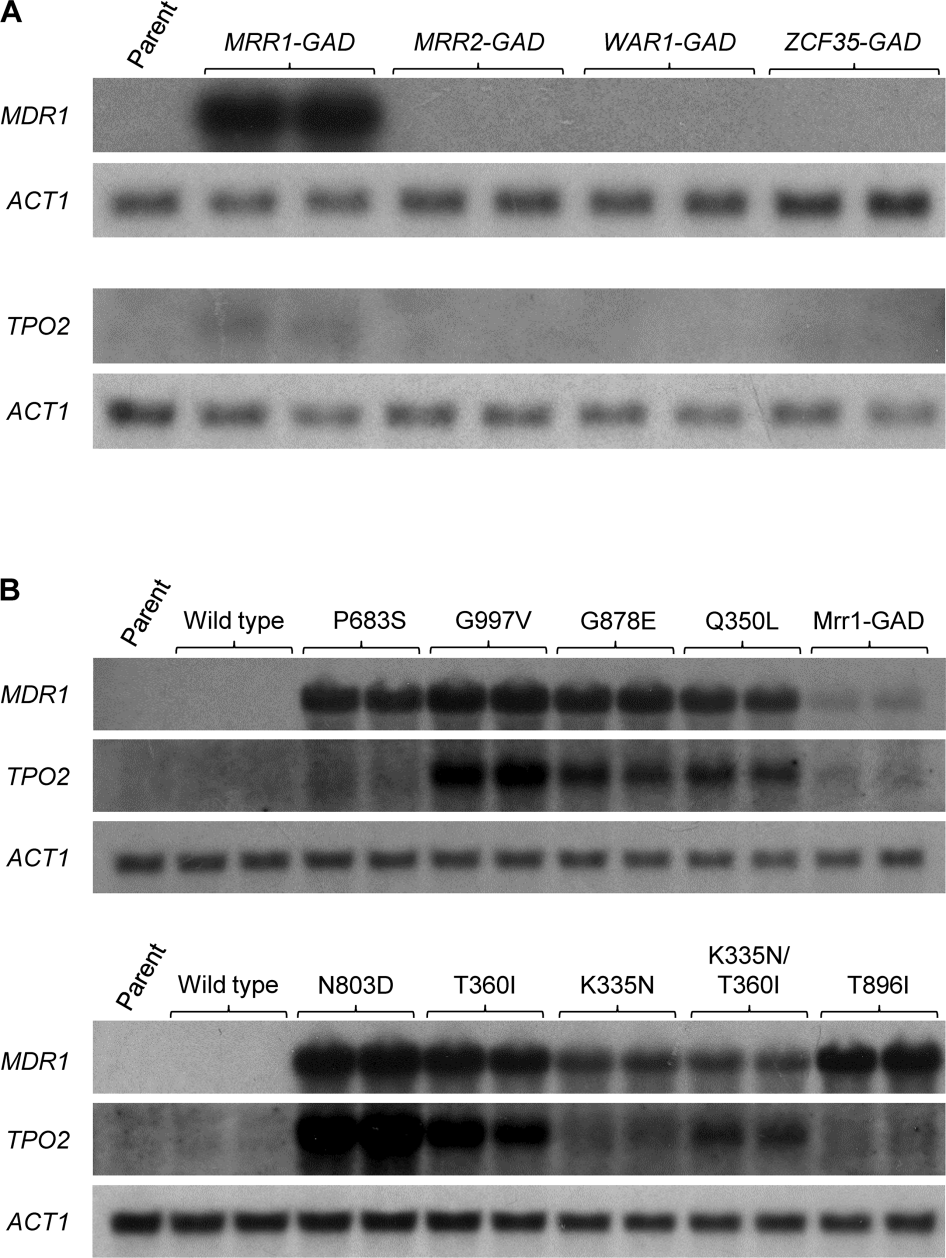
Analysis of *MDR1* and *TPO2* expression in strains containing hyperactive zinc cluster transcription factors. Strains were grown to log phase in YPD medium and gene expression was analyzed by Northern hybridization with specific probes. The *ACT1* transcript served as control for equal loading on each blot. (A) Strains expressing artificially activated *MRR1*, *MRR2*, *WAR1*, and *ZCF35*: SC5314 (Parent), SCMRR1GAD1A and -B (*MRR1-GAD*), SCZCF34GAD1A and -B (*MRR2-GAD*), SCWAR1GAD1A and -B (*WAR1-GAD*), SCZCF35GAD1A and -B (*ZCF35-GAD*). (B) Strains containing GOF mutations in Mrr1: SC5314 (Parent), SCMRR1R24A and -B (Wild type), SCMRR1R34A and -B (P683S), SCMRR1R44A and -B (G997V), SCMRR1R54A and -B (G878E), SCMRR1R64A and -B (Q350L), SCMRR1R74A and -B (N803D), SCMRR1R84A and -B (T360I), SCMRR1R94A and -B (K335N), SCMRR1R982A and -B (K335N/T360I), SCMRR1R104A and -B (T896I). Strains SCMRR1GAD1A and -B (Mrr1-GAD) were included for comparison.

As hyperactive forms of Mrr1 cause overexpression of many genes [16, 45], we searched the known Mrr1 target genes for candidates that might promote Hst 5 resistance. *TPO2* encodes another putative efflux pump that is closely related to Flu1 [41], and it is bound and upregulated by a hyperactive Mrr1 [45]. Mdr1 also has high similarity to Flu1 [42], and *MDR1* is one of the most strongly upregulated genes in strains with Mrr1 GOF mutations [16, 45]. Although no role of *TPO2* and *MDR1* in Hst 5 resistance had been found in a previous study [41], it seemed possible that these Mrr1 target genes could mediate Hst 5 resistance when they are overexpressed. Northern hybridization experiments showed that both *MDR1* and *TPO2* are expressed only at low levels in the wild-type strain SC5314 (Fig 6, left lanes). Interestingly, only Mrr1, but not the other artificially activated transcription factors, Mrr2, War1, and Zcf35, caused upregulation of *MDR1* and, more weakly, *TPO2* (Fig 6A), providing a potential explanation why the latter did not cause Hst 5 resistance. Most naturally occurring GOF mutations in Mrr1 had an even stronger effect on *MDR1* and *TPO2* expression than the artificially activated Mrr1, although expression levels strongly depended on the specific allele (Fig 6B).

**Fig 7:**
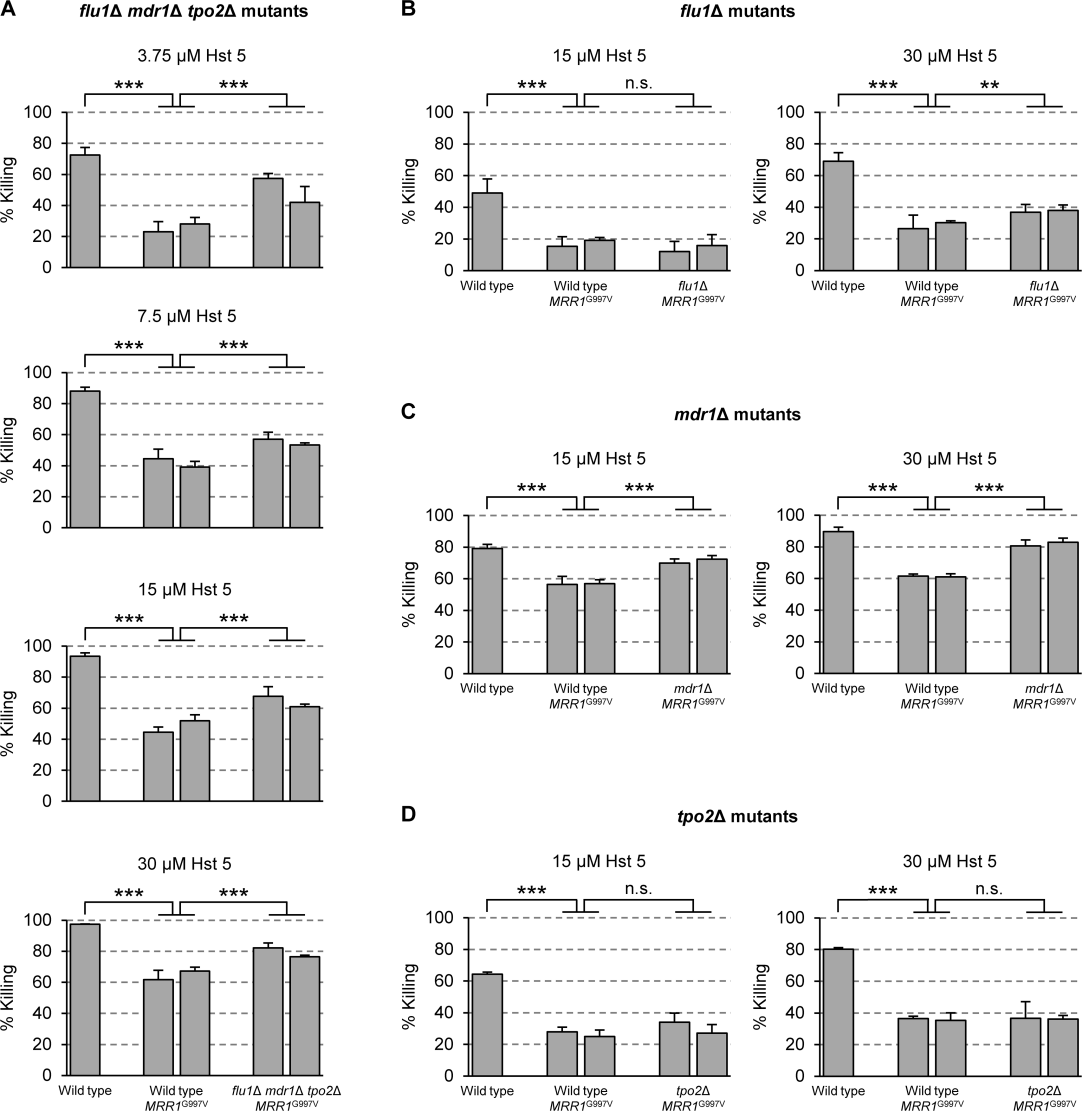
Mrr1-mediated Hst 5 resistance is mainly caused by *MDR1* overexpression. Strains were incubated in the presence of the indicated Hst 5 concentrations and the percent killing was determined as described in Materials and Methods. The different mutants were tested on separate occasions and compared with the wild-type controls. (A) Hst 5 sensitivity of strains containing the hyperactive *MRR1*^G997V^ allele in wild-type and *flu1*Δ *mdr1*Δ *tpo2*Δ triple mutant backgrounds. (B-D) Hst 5 sensitivity of strains containing the hyperactive *MRR1*^G997V^ allele in wild-type and (B) *flu1*Δ, (C) *mdr1*Δ, and (D) *tpo2*Δ single mutant backgrounds. The following strains were used in these experiments: SC5314 (Wild type), SCMRR1R44A and -C (Wild type, *MRR1*^G997V^), SC∆*flu1*∆*mdr1*∆*tpo2*MRR1R44A and -B (*flu1*Δ *mdr1*Δ *tpo2*Δ, *MRR1*^G997V^), SCΔ*flu1*MRR1R44A and -B (*flu1*Δ, *MRR1*^G997V^), SCΔ*mdr1*MRR1R44C and -D (*mdr1*Δ, *MRR1*^G997V^), SCΔ*tpo2*MRR1R44A and -B (*tpo2*Δ, *MRR1*^G997V^). Significant differences (ANOVA) are marked with asterisks (** *P* < 0.01, *** *P* < 0.001); n.s., not significant (*P* > 0.05).

As *FLU1*, *MDR1*, and *TPO2* all are upregulated by a hyperactive Mrr1, it was conceivable that each of these three related transporters contributes to Mrr1-mediated Hst 5 resistance and the importance of a single efflux pump would not be easily detectable. We therefore constructed *flu1*∆ *mdr1*∆ *tpo2*∆ triple mutants and introduced the G997V GOF mutation, which caused an efficient upregulation of all three genes (see Fig 4A and Fig 6B), into both *MRR1* alleles of the mutants. The deletion of the transporters in the wild-type parental strain, in which they were not or only weakly expressed, did not result in hypersusceptibility to Hst 5 (S4 Fig). However, the increased Hst 5 resistance conferred by the hyperactive Mrr1 was reduced, albeit not abolished, in the triple mutants, indicating that one or more of the transporters, but also additional Mrr1 target genes, promote Hst 5 resistance (Fig 7A). To assess the relative contribution of each individual transporter, we introduced the G997V mutation also into both *MRR1* alleles of *flu1*∆, *mdr1*∆, and *tpo2*∆ single mutants. Deletion of *FLU1* or *TPO2* did not or only slightly reduce the Hst 5 resistance of cells containing the *MRR1*^G997V^ mutation (Fig 7B and 7D). In contrast, deletion of *MDR1* increased the Hst 5 susceptibility of cells with the hyperactive Mrr1 (killing increased from *ca*. 60% to *ca*. 80% in the presence of 30 μM Hst 5), although the cells were still more resistant than the wild type (*ca*. 90% killing) (Fig 7C). These results demonstrated that overexpression of *MDR1* is one mechanism that contributes to the increased Hst 5 resistance of cells with Mrr1 GOF mutations.

### Fluconazole-resistant *C*. *albicans* isolates with Mrr1 GOF mutations have acquired increased Hst 5 resistance

We next investigated if fluconazole-resistant clinical *C*. *albicans* isolates that have acquired *MRR1* GOF mutations during antimycotic therapy also exhibit increased Hst 5 resistance. For this purpose, we selected five matched pairs of fluconazole-susceptible and -resistant isolates from AIDS patients with different *MRR1* GOF mutations. We had previously generated *mrr1*∆ mutants from the five resistant isolates [13, 16], which allowed us to assess the contribution of Mrr1 to their phenotypes. To compare *FLU1* expression levels, we introduced the P_*FLU1*_*-GFP* reporter fusion into all these strains. Two independent reporter strains were derived from each clinical isolate and one from both independently constructed *mrr1*∆ mutants of each resistant isolate to ensure the reproducibility of the results (one of the *mrr1*∆ mutants of isolate 2271 exhibited a high unspecific autofluorescence and could not be used for these experiments). As can be seen in S5 Fig, *FLU1* expression was moderately increased in all five resistant isolates compared to the matched susceptible isolates (2- to 5-fold), and the elevated expression levels returned to those observed in the susceptible isolates when *MRR1* was deleted. These and previous results [13, 16] demonstrate that Mrr1 GOF mutations cause concomitant upregulation of both efflux pumps, *MDR1* and *FLU1*, in fluconazole-resistant clinical *C*. *albicans* isolates.

**Fig 8:**
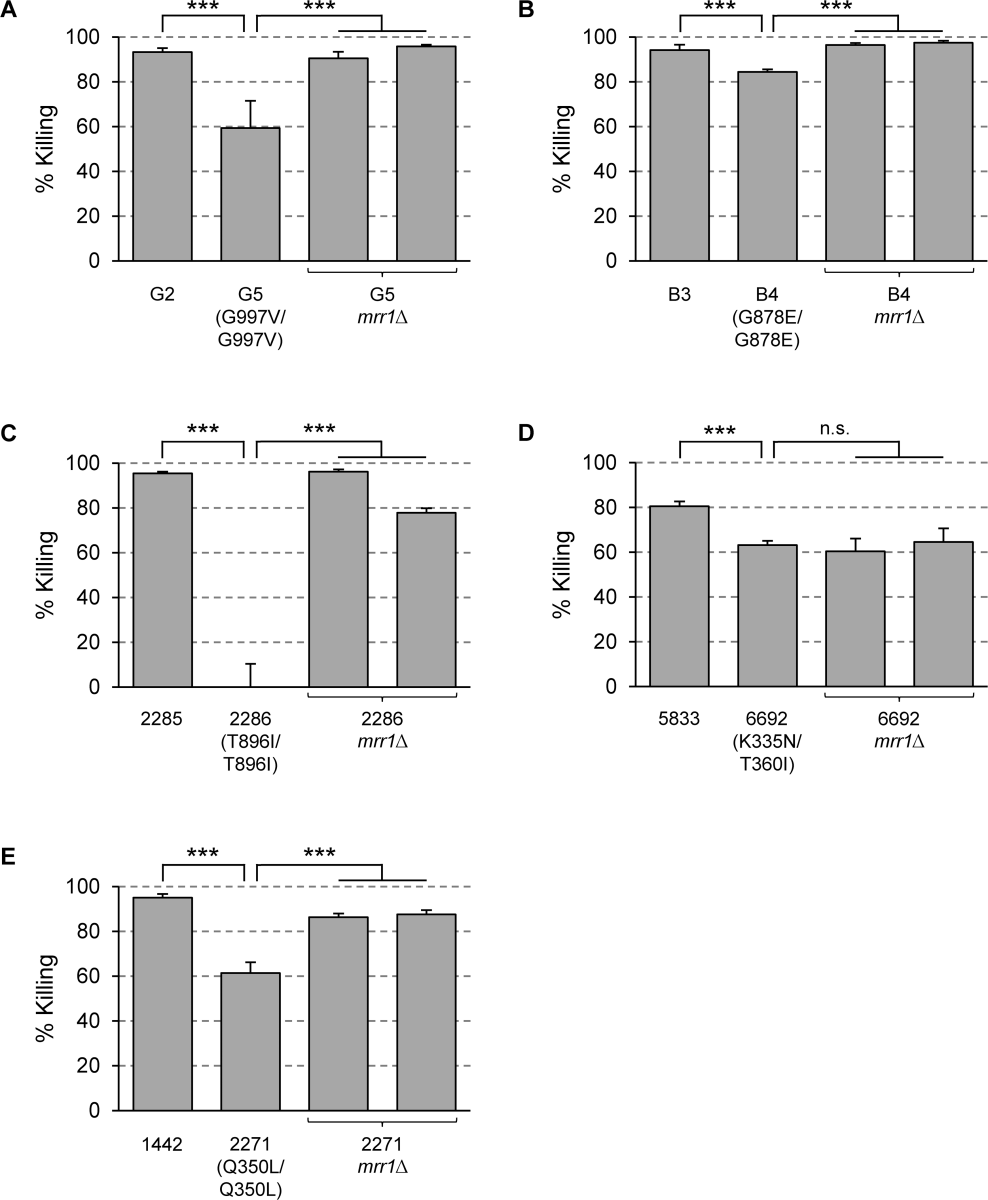
Clinical *C*. *albicans* isolates with Mrr1 GOF mutations exhibit increased Hst 5 resistance. Strains were incubated in the absence or presence of 30 μM Hst 5 and the percent killing was determined as described in Materials and Methods. (A)-(E) show the results obtained with five different sets of fluconazole-susceptible isolates, matched fluconazole-resistant isolates from the same patients containing the indicated Mrr1 GOF mutations, and *mrr1*∆ mutants derived from the resistant isolates. The following strains were used in these experiments: G2, G5, G5MRR1M4A and -B (G5*mrr1*∆), B3, B4, B4MRR1M4A and -B (B4*mrr1*∆), DSY2285 (2285), DSY2286 (2286), DSY2286MRR1M4A and -B (2286*mrr1*∆), 5833, 6692, 6692MRR1M4A and -B (6692*mrr1*∆), 1442, 2271, 2271MRR1M4A and -B (2271*mrr1*∆). Significant differences (ANOVA) are marked with asterisks (*** *P* < 0.001); n.s., not significant (*P* > 0.05).

We then assessed if the Mrr1 GOF mutations also resulted in enhanced Hst 5 resistance of the clinical isolates. Fig 8 shows that killing by Hst 5 was reduced in all five fluconazole-resistant isolates with Mrr1 GOF mutations compared to their matched susceptible isolates, but to different degrees. A relatively minor but significant reduction in killing (from 94% to 84%) was observed for isolate B4 compared to isolate B3 (Fig 8B), whereas isolate DSY2286 was highly resistant, as hardly any killing of this isolate was observed at the tested Hst 5 concentration (Fig 8C). In four of the five cases, Hst 5 resistance was fully or largely mediated by the hyperactive Mrr1, because the percent killing was elevated to the levels of the matched susceptible isolates when *MRR1* was deleted. Isolate 6692 was an exception, because the *mrr1*∆ mutants derived from it retained the increased Hst 5 resistance. This strain apparently possesses other mechanisms of Hst 5 resistance that override the contribution of the hyperactive Mrr1. Collectively, these results demonstrate that fluconazole-resistant *C*. *albicans* strains with Mrr1 GOF mutations have simultaneously acquired increased Hst 5 resistance, because the overexpression of *MDR1* and other Mrr1 target genes not only mediates fluconazole resistance but also increased resistance to the antimicrobial peptide.

## Discussion

The acquisition of GOF mutations in zinc cluster transcription factors is a frequent cause of fluconazole resistance in clinical *C*. *albicans* strains. GOF mutations in Mrr1, Tac1, and Upc2 enable the cells to continue to grow in the presence of the drug and outcompete wild-type cells in the population. However, the hyperactive transcription factors also decrease the fitness of the cells in the absence of drug selection, at least under some environmental conditions [20, 44, 46]. As Mrr1, Tac1, and Upc2 regulate different sets of genes [31, 45, 47], the fitness defect must have a different basis in each case; consequently, it is aggravated in highly resistant strains containing several hyperactive transcription factors [20]. The deregulated gene expression probably causes an unnecessary waste of energy and reduces the ability of the cells to appropriately adapt to specific host niches. Here, we have uncovered a previously unrecognized effect of hyperactive forms of Mrr1 in addition to their ability to mediate fluconazole resistance. The overexpression of the multidrug efflux pump *MDR1* (and possibly to some extent also *FLU1*) and other Mrr1 target genes confers increased resistance to the antifungal peptide Hst 5, which is present in the saliva of humans. Fluconazole-resistant strains with GOF mutations in Mrr1 have mainly been isolated from the oral cavity of HIV-infected patients suffering from oropharyngeal candidiasis [13, 14, 16, 42, 44, 48–55]. Increased Hst 5 resistance may therefore represent an additional advantage that counterbalances the fitness costs of Mrr1 hyperactivity and helps the mutants to establish themselves in this host niche. Nevertheless, Mrr1 mutations have been found only after fluconazole treatment of oropharyngeal candidiasis and have not been detected in pretreatment isolates from the same patients. This suggests that exposure to Hst 5 is not sufficient to select for Mrr1 GOF mutations when *C*. *albicans* colonizes the oral cavity as a harmless commensal without causing disease symptoms and does not have to cope with antimycotic therapy.

It is difficult to estimate how strongly a hyperactive Mrr1 contributes to the survival and competitive fitness of *C*. *albicans* in the oral cavity of humans. After short-term exposure to Hst 5, as in our *in vitro* experiments, strains with a hyperactive Mrr1 exhibited significantly reduced killing compared to wild-type controls. In the natural habitat, Hst 5 is constantly produced, resulting in continued exposure of *C*. *albicans* to the antifungal peptide, and the ability to avoid its toxic effects may present an even more significant advantage. The fitness gain due to enhanced Hst 5 resistance will likely also depend on the Hst 5 concentration, which varies among individuals and is generally lower in HIV-infected persons [56]. As Hst 5 is produced only by humans and other primates, the relevance of increased Hst 5 resistance cannot be meaningfully studied in mouse models of experimental candidiasis.

Although we initially searched for regulators of *FLU1* expression in order to identify transcription factors that might confer Hst 5 resistance, our subsequent experiments revealed that Flu1 contributed little, if at all, to Hst 5 resistance in derivatives of strain SC5314 carrying hyperactive forms of *MRR1*. Instead, we unexpectedly found that overexpression of the efflux pump *MDR1* was one mechanism by which such strains became less susceptible to killing by the antimicrobial peptide. *MDR1* overexpression, therefore, confers increased resistance not only to fluconazole but also to Hst 5, which is a previously unrecognized function of this efflux pump that is of potential clinical relevance. It is well possible that *FLU1* makes a more important contribution in some clinical isolates with *MRR1* GOF mutations, because all such isolates that were tested concomitantly overexpressed both *MDR1* and *FLU1*, and some of them may contain *FLU1* alleles that encode more efficient Hst 5 transporters. Allelic differences that affect the efficiency of drug transport have been previously reported for the drug efflux pump *CDR2* [57]. Nevertheless, a hyperactive Mrr1 still conferred increased Hst 5 resistance, albeit less efficiently, even in strains lacking both *MDR1* and *FLU1* as well as *TPO2*, encoding a related putative transporter, indicating that additional Mrr1 target genes are involved in this phenotype. This is similar to Mrr1-mediated fluconazole resistance, which is only partially mediated by Mdr1 and involves other Mrr1 target genes that remain to be identified [45]. The effect of specific GOF mutations on the expression of individual Mrr1 target genes varies. For example, the T360I and T896I mutations caused comparable *MDR1* expression levels, but only the T360I mutation also resulted in strong *TPO2* overexpression (see Fig 6B). Therefore, the degree of resistance to fluconazole and Hst 5 conferred by a particular Mrr1 GOF mutation will depend not only on the expression levels of *MDR1* but also on those of the other target genes that contribute to these phenotypes.

The successful establishment and expansion of genetically altered variants within a population require that the beneficial effects of a mutation outweigh its negative consequences. As noted above, the increased Hst 5 resistance may not sufficiently offset the fitness costs caused by a hyperactive Mrr1 to select for *MRR1* GOF mutations in strains colonizing the oral cavity of healthy persons. In contrast, the increased fluconazole resistance of such strains supports their emergence in the presence of the drug. It is intriguing that antimycotic therapy promotes the evolution of strains which, as a consequence of a drug resistance mutation, have acquired increased resistance to an innate host defense mechanism and are better adapted to some stressful conditions encountered in the host even in the absence of antifungal drug treatment.

## Materials and methods

### Strains and growth conditions

*Candida albicans* strains used in this study are listed in S1 Table. The complete library of *C*. *albicans* strains containing artificially activated zinc cluster transcription factors has been described previously [34]. All strains were stored as frozen stocks with 17.2% glycerol at -80°C and subcultured on YPD agar plates (10 g yeast extract, 20 g peptone, 20 g glucose, 15 g agar per litre) at 30°C. Strains were routinely grown in YPD liquid medium at 30°C in a shaking incubator. For selection of nourseothricin-resistant transformants, 200 μg/ml nourseothricin (Werner Bioagents, Jena, Germany) was added to YPD agar plates. To obtain nourseothricin-sensitive derivatives in which the *SAT1* flipper cassette was excised by FLP-mediated recombination, transformants were grown overnight in YCB-BSA-YE medium (23.4 g yeast carbon base, 4 g bovine serum albumin, 2 g yeast extract per litre, pH 4.0) without selective pressure to induce the *SAP2* promoter controlling *caFLP* expression. Alternatively, strains containing a *SAT1* flipper cassette in which the *caFLP* gene is expressed from the *MAL2* promoter (as in plasmids pMRR1R4 to pMRR1R10) were grown overnight in YPM medium (10 g yeast extract, 20 g peptone, 20 g maltose per liter) instead of YCB-BSA-YE to induce the *MAL2* promoter. Appropriate dilutions of the cultures were plated on YPD agar plates and grown for 2 days at 30°C to obtain single colonies. Nourseothricin-sensitive clones were identified by restreaking on YPD plates and on YPD plates containing 100 μg/ml nourseothricin.

### Plasmid constructions

A *FLU1* deletion construct was obtained by amplification of the *FLU1* upstream and downstream regions from genomic DNA of strain SC5314 with the primer pairs FLU1.01/FLU1.02 and FLU1.03/FLU1.04, respectively (all oligonucleotide primers used in this study are listed in S2 Table). The PCR products were digested with SacI/SacII and XhoI/ApaI, respectively, and cloned on both sides of the modified *SAT1* flipper cassette contained in plasmid pSFS5 [58] to result in pFLU1M1. To reintroduce an intact *FLU1* copy into *flu1*∆ mutants, the *FLU1* coding region plus upstream and downstream sequences was amplified with primers FLU1.01 and FLU1_compleR. The PCR product was digested with SacI/SacII and substituted for the *FLU1* upstream region of plasmid pFLU1M1 to generate pFLU1K1. A P_*FLU1*_*-GFP* reporter fusion was constructed as follows. The *FLU1* upstream region was amplified with primers FLU1.01 and FLU1rev_upstream, and a *GFP*-T_*ACT1*_ fragment was amplified from plasmid pNIM1 [59] with primers FLU1forw_GFP and ACT19. The gel-purified PCR products were then used as templates for a fusion PCR with primers FLU1.01 and ACT19. The PCR product was digested with SacI/SacII and inserted instead of the *FLU1* upstream region in plasmid pFLU1M1 to generate pFLU1G1.

A *WAR1* deletion construct was generated by amplifying the *WAR1* flanking sequences with the primer pairs WAR1.01/WAR1.02 and WAR1.03/WAR1.04, digesting the PCR products with SacI/SacII and XhoI/ApaI, respectively, and inserting the fragments on both sides of the *SAT1* flipper cassette of pSFS5, generating pWAR1M1. Similarly, a *ZCF35* deletion cassette was obtained by amplifying the *ZCF35* flanking sequences with the primer pairs ZCF35-7/ZCF35-8 and ZCF35-9/ZCF35-10 and cloning the SacI/SacII- and XhoI/ApaI-digested PCR products in pSFS5 to generate pZCF35M3. A *TPO2* deletion cassette was generated by amplifying the *TPO2* flanking sequences with the primer pairs TPO2.01/TPO2.02 and TPO2.03/TPO2.06 and cloning the SacI/SacII- and XhoI/KpnI-digested PCR products in pSFS5 to generate pTPO2M2. A new *MDR1* deletion cassette was generated by substituting the *SAT1* flipper cassette from pSFS5 for the old *SAT1* flipper cassette in the previously described plasmid pMDR1M2 [45], yielding pMDR1M3.

Plasmids pMRR1R2 and pMRR1R3, which contain the wild-type *MRR1* gene and a mutated allele with the P683S GOF mutation, respectively, have been previously described [20, 45]. These plasmids also contain the recyclable *SAT1* flipper cassette to allow the sequential replacement of both endogenous *MRR1* alleles in strain SC5314. To obtain analogous constructs with other *MRR1* GOF mutations, the SacI-BglII fragments from plasmids pZCF36K5, pZCF36K12, pZCF36K14, pZCF36K15, pZCF36K17, pZCF36K18, and pZCF36K19, containing G997V, G878E, Q350L, N803D, T360I, K335N, and T896I GOF mutations, respectively, in *MRR1* [13], were inserted instead of the wild-type *MRR1*, generating plasmids pMRR1R4 to pMRR1R10.

### Strain constructions

*C*. *albicans* strains were transformed by electroporation [43] with the following gel-purified linear DNA fragments. The insert from pFLU1M1 was used to sequentially delete the two wild-type alleles of *FLU1* in strain SC5314, and the insert from pFLU1K1 was used to reintroduce an intact *FLU1* copy into the homozygous mutants. The insert from pFLU1G1 was used to express the *GFP* reporter gene under the control of the endogenous *FLU1* promoter in various strain backgrounds. The cassettes from plasmids pMRR1GAD1, pZCF34GAD1, pWAR1GAD1, and pZCF35GAD1 [34] were used to introduce artificially activated forms of *MRR1*, *MRR2*, *WAR1*, and *ZCF35*, respectively, into P_*FLU1*_*-GFP* reporter strains and *flu1*∆ mutants. The deletion cassettes from pMDR1M3, pTPO2M2, pWAR1M1, and pZCF35M3 were used to generate *mdr1*∆, *tpo2*∆, *war1*∆, and *zcf35*∆ mutants of strain SC5314. The cassettes from pMDR1M3 and pTPO2M2 were also used for the construction of *flu1*∆ *mdr1*∆ *tpo2*∆ triple mutants. The inserts from plasmids pMRR1R4 to pMRR1R10 were used to replace the wild-type *MRR1* alleles of strain SC5314 by mutated alleles with different GOF mutations. The insert from pMRR1R4 was also used to introduce the G997V GOF mutation into both *MRR1* alleles of *flu1*∆, *mdr1*∆, and *tpo2*∆ single mutants and *flu1*∆ *mdr1*∆ *tpo2*∆ triple mutants. The correct integration of each construct as well as recycling of the *SAT1* flipper cassette were confirmed by Southern hybridization using the flanking sequences as probes. Introduction of the *MRR1* GOF mutations was confirmed by reamplification of the genes from heterozygous and homozygous mutants and sequencing of the PCR products. In each case, two independent series of strains were generated and used for further analysis.

### Isolation of genomic DNA and Southern hybridization

Genomic DNA from *C*. *albicans* strains was isolated as described previously [60]. The DNA was digested with appropriate restriction enzymes, separated on a 1% agarose gel, transferred by vacuum blotting onto a nylon membrane, and fixed by UV crosslinking. Southern hybridization with enhanced chemiluminescence-labeled probes was performed with the Amersham ECL Direct Nucleic Acid Labelling and Detection System (GE Healthcare UK Limited, Little Chalfont Buckinghamshire, UK) according to the instructions of the manufacturer.

### Northern hybridization analysis

Overnight cultures of the strains were diluted 10^−2^ in fresh YPD medium and grown for 4 h at 30°C. Total RNA was extracted by the hot acidic phenol method [61] combined with a purification step with the RNeasy Mini Kit (Qiagen, Hilden, Germany). RNA samples were separated on a 1.2% agarose gel, transferred by capillary blotting onto a nylon membrane, fixed by UV crosslinking, and hybridized with digoxigenin-labeled *MDR1* (positions 647 to 1688 in the *MDR1* coding sequence, amplified with primers NB-MDR1_FW and NB-MDR1-RV), *TPO2* (positions 115 to 1154 in the *TPO2* coding sequence, amplified with primers TPO2_forN3 and TPO3_revN3), and *ACT1* (positions 1512 to 1677 in the *ACT1* coding sequence, amplified with primers ACT_RT and ACT2_RT) probes. The bound probes were detected with a peroxidase-labeled anti-digoxigenin AP-conjugate (Roche, Basel, Switzerland).

### Fluconazole susceptibility assays

The fluconazole susceptibilities of the strains were determined by a previously described broth microdilution method [62], with slight modifications. A 2-day-old colony from a YPD agar plate was suspended in 2 ml of a 0.9% NaCl solution, and 4 μl of the suspension was mixed with 2 ml 2x SD-CSM medium (13.4 g yeast nitrogen base without amino acids [YNB; BIO 101, Vista, Calif.], 40 g glucose, 1.54 g complete supplement medium [CSM, BIO101]). A twofold dilution series of fluconazole (Sigma GmbH, Deisenhofen, Germany) was prepared in water, starting from an initial concentration of 512 μg/ml. One hundred microliters of each fluconazole solution was then mixed with 100 μl of the cell suspension in a 96-well microtiter plate and the plates were incubated for 48 h at 37°C. The MIC of fluconazole was defined as the drug concentration that abolished or drastically reduced visible growth compared to a drug-free control.

### Dilution spot assays

Overnight cultures of the strains in SD-CSM medium were diluted to an optical density at 600 nm of 2.0. Ten-fold dilutions from 10^0^ to 10^−5^ were prepared in a 96-well microtiter plate and *ca*. 5 μl of the cell suspensions transferred with a replicator onto SD-CSM agar plates without or with 0.5 or 1.0 μg/ml MPA or 5 μg/ml fluconazole. Plates were incubated for 2 days at 30°C and photographed.

### Flow cytometry

Overnight cultures of the *GFP* reporter and control strains were diluted 10^−2^ in fresh YPD medium and grown for 3 h at 30°C. The cultures were tenfold diluted in 1 ml cold phosphate-buffered saline (PBS) and flow cytometry was performed using the MACSQuantAnalyzer (Miltenyi Biotec; Bergisch Gladbach, Germany) equipped with an argon laser emitting at 488 nm. Fluorescence was detected using the B1 fluorescence channel equipped with a 525 nm band-pass filter (bandwidth 50 nm). Twenty thousand cells were analyzed per sample and counted at a flow rate of approx. 500 cells per second. Fluorescence data were collected by using logarithmic amplifiers. The mean fluorescence (arbitrary values) was determined with MACSQuantify (Version 2.4, Miltenyi Biotec) software.

### Hst 5 sensitivity assays

The susceptibility of *C*. *albicans* cells to Hst 5 was measured using microdilution plate assays as previously described [63]. Briefly, 10 ml of YPD medium was inoculated with single colonies of each strain. Cells were grown overnight at room temperature. Overnight cultures were diluted to an *A*_600_ of 0.3 to 0.4 and then incubated at 30°C with shaking (220 rpm) until an *A*_600_ of ∼1.0 was reached. Cells were washed twice with 10 mM sodium phosphate buffer (NaPB), pH 7.4. The cells (1 × 10^6^) were then mixed with different concentrations of Hst 5 at 30°C for 60 min and diluted in 10 mM NaPB. Aliquots of 500 cells were spread onto YPD agar plates and incubated for 24 to 48 h until colonies became visible. The percent killing was calculated as [1 − (number of colonies from Hst 5-treated cells/number of colonies from control cells)] × 100%. Assays were performed in quadruplicate for each strain. The reference strain SC5314 was included in each set of experiments to validate assay conditions.

### Statistical analysis

Since the *FLU1* expression and Hst 5 sensitivity assays were performed under controlled conditions *in vitro*, we assumed that the collected data fit a standard normal distribution. The one-way ANOVA test was used when three or more groups were compared with each other, and the two-tailed t-test was used when only two groups were compared, as indicated in the figures. Values for the two independently constructed strains A and B were combined in each case, except for the experiments shown in Fig 4B, which were performed on separate occasions. All statistical tests were conducted using GraphPad Prism version 7.03 software.

## Supporting information

S1 Fig MPA sensitivity of *flu1*∆ mutants. Serial dilutions of the wild type and two independent series of heterozygous and homozygous *flu1*∆ mutants and complemented strains were spotted onto SD-CSM agar plates without or with 0.5 μg/ml MPA and grown for 2 days at 30°C. The following strains were used in this experiment: SC5314 (Wild type), SCFLU1M2A and -B (*FLU1*/*flu1*∆), SCFLU1M4A and -B (*flu1*∆/*flu1*∆), SCFLU1K2A and -B (*flu1*∆/*flu1*∆ + *FLU1*). Strain S2UI1, which carries a mutated *IMH3* allele that confers MPA resistance (*MPA*^R^), was included as an additional control.
(PDF)

S2 Fig *FLU1* expression in strains carrying a P_*FLU1*_*-GFP* reporter fusion in the indicated genetic backgrounds. Strains were grown to log phase in YPD medium and the mean fluorescence of the cells was determined by flow cytometry. The results obtained with two independently generated reporter strains are shown in each case (means and standard deviations from three biological replicates). The background fluorescence of otherwise identical strains without *GFP* is indicated by the black part of each column. *GFP* reporter strains: SCFLU1G2A and -B (Wild type), SCMRR1M4FLU1G2A and -B (*mrr1*∆), SCMRR2M4FLU1G2A and -B (*mrr2*∆), SCWAR1M4FLU1G2A and -B (*war1*∆), SCZCF35M4FLU1G2A and -B (*zcf35*∆). Control strains without *GFP*: SC5314 (Wild type), SCMRR1M4A and -B (*mrr1*∆), SCZCF34M4A and -B (*mrr2*∆), SCWAR1M4A and -B (*war1*∆), SCZCF35M4A and -B (*zcf35*∆). n.s., not significantly different from wild-type control (*P* > 0.05, ANOVA).
(PDF)

S3 Fig Replacement of the endogenous *MRR1* alleles of strain SC5314 with two nonmutated wild-type copies does not affect Hst 5 susceptibility. Strains were incubated in the absence or presence of 30 μM Hst 5 and the percent killing was determined as described in Materials and Methods. The following strains were used in this experiment: SC5314 (Parent), SCMRR1R24A (Wild type A), SCMRR1R24B (Wild type B). n.s., not significantly different from parent (*P* > 0.05, t-test).
(PDF)

S4 Fig Deletion of *FLU1*, *MDR1*, and *TPO2* in a wild-type background does not result in hypersensitivity to Hst 5. Strains were incubated in the absence or presence of 15 μM or 30 μM Hst 5 and the percent killing was determined as described in Materials and Methods. The following strains were used: SC5314 (Wild type), SCΔ*flu1*∆*mdr1*TPO2M4A and -B (*flu1*Δ *mdr1*Δ *tpo2*Δ). n.s., not significantly different from wild-type control (*P* > 0.05, t-test).
(PDF)

S5 Fig *FLU1* expression in the fluconazole-susceptible clinical isolates G2, B3, DSY2285 (2285), 5833, and 1442, the matched resistant isolates G5, B4, DSY2286 (2286), 6692, and 2271 containing the indicated *MRR1* GOF mutations, and two independent *mrr1*∆ mutants derived from each resistant isolate (only one *mrr1*∆ mutant for isolate 2271). A P_*FLU1*_*-GFP* reporter fusion was integrated into the strains (two independent transformants of each clinical isolate were used) and the mean fluorescence of cells grown to log phase in YPD medium was determined by flow cytometry (means and standard deviations from at least three biological replicates). The background fluorescence of the parental strains without *GFP* is indicated by the black part of the columns. *GFP* reporter strains: G2FLU1G2A and -B (G2), G5FLU1G2A and -B (G5), G5MRR1M4FLU1G2A and -B (G5*mrr1*∆), B3FLU1G2A and -B (B3), B4FLU1G2A and -B (B4), B4MRR1M4FLU1G2A and -B (B4*mrr1*∆), DSY2285FLU1G2A and -B (2285), DSY2286FLU1G2A and -B (2286), DSY2286MRR1M4FLU1G2A and -B (2286*mrr1*∆), 5833FLU1G2A and -B (5833), 6692FLU1G2A and -B (6692), 6692MRR1M4FLU1G2A and -B (6692*mrr1*∆), 1442FLU1G2A and -B (1442), 2271FLU1G2A and -B (2271), 2271MRR1M4FLU1G2A (2271*mrr1*∆). Control strains without *GFP*: G2, G5, G5MRR1M4A and -B (G5*mrr1*∆), B3, B4, B4MRR1M4A and -B (B4*mrr1*∆), DSY2285 (2285), DSY2286 (2286), DSY2286MRR1M4A and -B (2286*mrr1*∆), 5833, 6692, 6692MRR1M4A and -B (6692*mrr1*∆), 1442, 2271, 2271MRR1M4A (2271*mrr1*∆). Significant differences (background-subtracted values, ANOVA) are marked with asterisks (*** *P* < 0.001).
(PDF)

S1 Table *C*. *albicans* strains used in this study. (XLSX)

S2 Table Primers used in this study. (XLSX)
