# Supplementary material for: An acquired mechanism of antifungal drug resistance simultaneously enables Candida albicans to escape from intrinsic host defenses
Source: PLoS Pathog. 2017 Sep 27;13(9):e1006655. doi: 10.1371/journal.ppat.1006655 (PMC5633205; doi:10.1371/journal.ppat.1006655)
Supplement: S1 Fig — Serial dilutions of the wild type and two independent series of heterozygous and homozygous flu1∆ mutants and complemented strains were spotted onto SD-CSM agar plates without or with 0.5 μg/ml MPA and grown for 2 days at 30°C. The following strains were used in this experiment: SC5314 (Wild type), SCFLU1M2A and -B (FLU1/flu1∆), SCFLU1M4A and -B (flu1∆/flu1∆), SCFLU1K2A and -B (flu1∆/flu1∆ + FLU1). Strain S2UI1, which carries a mutated IMH3 allele that confers MPA resistance (MPAR), was included as an additional control. (PDF) [file ppat.1006655.s001.pdf]

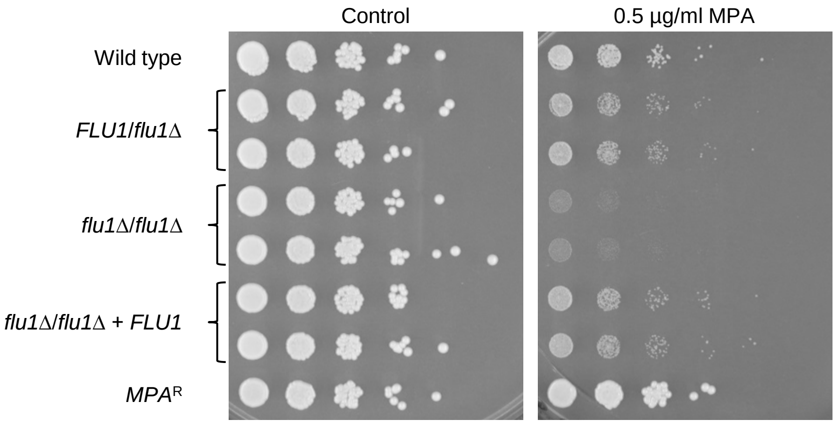

Figure S1 MPA sensitivity of *flu1*Δ mutants. Serial dilutions of the wild type and two independent series of heterozygous and homozygous *flu1*Δ mutants and complemented strains were spotted onto SD-CSM agar plates without or with 0.5 µg/ml MPA and grown for 2 days at 30°C. The following strains were used in this experiment: SC5314 (Wild type), SCFLU1M2A and -B (*FLU1*/*flu1*Δ), SCFLU1M4A and -B (*flu1*Δ/*flu1*Δ), SCFLU1K2A and -B (*flu1*Δ/*flu1*Δ + *FLU1*). Strain S2UI1, which carries a mutated *IMH3* allele that confers MPA resistance (*MPA*^{R}), was included as an additional control.
