# Supplementary material for: An acquired mechanism of antifungal drug resistance simultaneously enables Candida albicans to escape from intrinsic host defenses
Source: PLoS Pathog. 2017 Sep 27;13(9):e1006655. doi: 10.1371/journal.ppat.1006655 (PMC5633205; doi:10.1371/journal.ppat.1006655)
Supplement: S2 Fig — Strains were grown to log phase in YPD medium and the mean fluorescence of the cells was determined by flow cytometry. The results obtained with two independently generated reporter strains are shown in each case (means and standard deviations from three biological replicates). The background fluorescence of otherwise identical strains without GFP is indicated by the black part of each column. GFP reporter strains: SCFLU1G2A and -B (Wild type), SCMRR1M4FLU1G2A and -B (mrr1∆), SCMRR2M4FLU1G2A and -B (mrr2∆), SCWAR1M4FLU1G2A and -B (war1∆), SCZCF35M4FLU1G2A and -B (zcf35∆). Control strains without GFP: SC5314 (Wild type), SCMRR1M4A and -B (mrr1∆), SCZCF34M4A and -B (mrr2∆), SCWAR1M4A and -B (war1∆), SCZCF35M4A and -B (zcf35∆). n.s., not significantly different from wild-type control (P > 0.05, ANOVA). (PDF) [file ppat.1006655.s002.pdf]

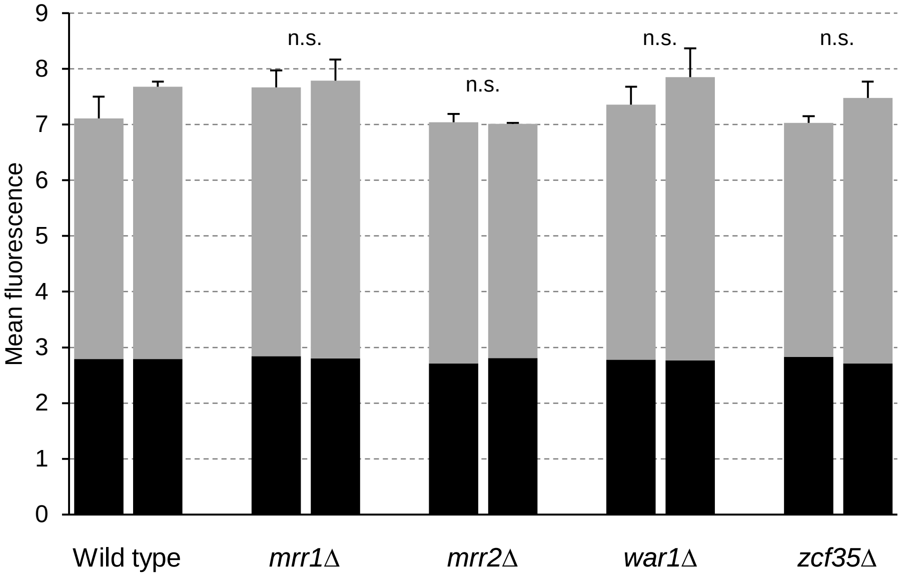


**Figure S2** *FLU1* expression in strains carrying a $P_{FLU1}$-*GFP* reporter fusion in the indicated genetic backgrounds. Strains were grown to log phase in YPD medium and the mean fluorescence of the cells was determined by flow cytometry. The results obtained with two independently generated reporter strains are shown in each case (means and standard deviations from three biological replicates). The background fluorescence of otherwise identical strains without *GFP* is indicated by the black part of each column. *GFP* reporter strains: SCFLU1G2A and -B (Wild type), SCMRR1M4FLU1G2A and -B (*mrr1*Δ), SCMRR2M4FLU1G2A and -B (*mrr2*Δ), SCWAR1M4FLU1G2A and -B (*war1*Δ), SCZCF35M4FLU1G2A and -B (*zcf35*Δ). Control strains without *GFP*: SC5314 (Wild type), SCMRR1M4A and -B (*mrr1*Δ), SCZCF34M4A and -B (*mrr2*Δ), SCWAR1M4A and -B (*war1*Δ), SCZCF35M4A and -B (*zcf35*Δ). n.s., not significantly different from wild-type control ($P > 0.05$, ANOVA).
