# Supplementary material for: An acquired mechanism of antifungal drug resistance simultaneously enables Candida albicans to escape from intrinsic host defenses
Source: PLoS Pathog. 2017 Sep 27;13(9):e1006655. doi: 10.1371/journal.ppat.1006655 (PMC5633205; doi:10.1371/journal.ppat.1006655)
Supplement: S3 Fig — Strains were incubated in the absence or presence of 30 μM Hst 5 and the percent killing was determined as described in Materials and Methods. The following strains were used in this experiment: SC5314 (Parent), SCMRR1R24A (Wild type A), SCMRR1R24B (Wild type B). n.s., not significantly different from parent (P > 0.05, t-test). (PDF) [file ppat.1006655.s003.pdf]

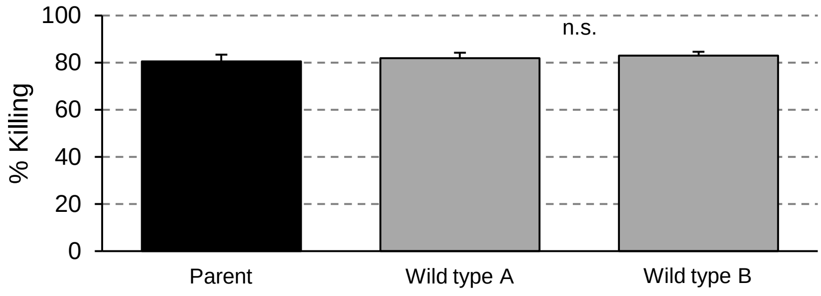

Figure S3 Replacement of the endogenous *MRR1* alleles of strain SC5314 with two nonmutated wild-type copies does not affect Hst 5 susceptibility. Strains were incubated in the absence or presence of 30 μM Hst 5 and the percent killing was determined as described in Materials and Methods. The following strains were used in this experiment: SC5314 (Parent), SCMRR1R24A (Wild type A), SCMRR1R24B (Wild type B). n.s., not significantly different from parent ($P > 0.05$, t-test).
