# Supplementary material for: An acquired mechanism of antifungal drug resistance simultaneously enables Candida albicans to escape from intrinsic host defenses
Source: PLoS Pathog. 2017 Sep 27;13(9):e1006655. doi: 10.1371/journal.ppat.1006655 (PMC5633205; doi:10.1371/journal.ppat.1006655)
Supplement: S4 Fig — Strains were incubated in the absence or presence of 15 μM or 30 μM Hst 5 and the percent killing was determined as described in Materials and Methods. The following strains were used: SC5314 (Wild type), SCΔflu1∆mdr1TPO2M4A and -B (flu1Δ mdr1Δ tpo2Δ). n.s., not significantly different from wild-type control (P > 0.05, t-test). (PDF) [file ppat.1006655.s004.pdf]

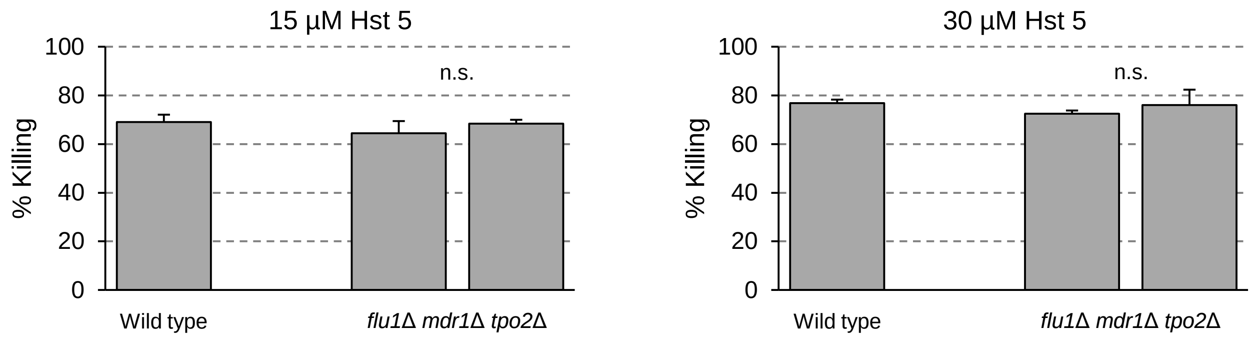

Figure S4 Deletion of *FLU1*, *MDR1*, and *TPO2* in a wild-type background does not result in hypersensitivity to Hst 5. Strains were incubated in the absence or presence of 15 µM or 30 µM Hst 5 and the percent killing was determined as described in Materials and Methods. The following strains were used: SC5314 (Wild type), SCΔ*flu1*Δ*mdr1*TPO2M4A and -B (*flu1*Δ *mdr1*Δ *tpo2*Δ). n.s., not significantly different from wild-type control ($P > 0.05$, t-test).
