# Supplementary material for: An acquired mechanism of antifungal drug resistance simultaneously enables Candida albicans to escape from intrinsic host defenses
Source: PLoS Pathog. 2017 Sep 27;13(9):e1006655. doi: 10.1371/journal.ppat.1006655 (PMC5633205; doi:10.1371/journal.ppat.1006655)
Supplement: S5 Fig — A PFLU1-GFP reporter fusion was integrated into the strains (two independent transformants of each clinical isolate were used) and the mean fluorescence of cells grown to log phase in YPD medium was determined by flow cytometry (means and standard deviations from at least three biological replicates). The background fluorescence of the parental strains without GFP is indicated by the black part of the columns. GFP reporter strains: G2FLU1G2A and -B (G2), G5FLU1G2A and -B (G5), G5MRR1M4FLU1G2A and -B (G5mrr1∆), B3FLU1G2A and -B (B3), B4FLU1G2A and -B (B4), B4MRR1M4FLU1G2A and -B (B4mrr1∆), DSY2285FLU1G2A and -B (2285), DSY2286FLU1G2A and -B (2286), DSY2286MRR1M4FLU1G2A and -B (2286mrr1∆), 5833FLU1G2A and -B (5833), 6692FLU1G2A and -B (6692), 6692MRR1M4FLU1G2A and -B (6692mrr1∆), 1442FLU1G2A and -B (1442), 2271FLU1G2A and -B (2271), 2271MRR1M4FLU1G2A (2271mrr1∆). Control strains without GFP: G2, G5, G5MRR1M4A and -B (G5mrr1∆), B3, B4, B4MRR1M4A and -B (B4mrr1∆), DSY2285 (2285), DSY2286 (2286), DSY2286MRR1M4A and -B (2286mrr1∆), 5833, 6692, 6692MRR1M4A and -B (6692mrr1∆), 1442, 2271, 2271MRR1M4A (2271mrr1∆). Significant differences (background-subtracted values, ANOVA) are marked with asterisks (*** P < 0.001). (PDF) [file ppat.1006655.s005.pdf]

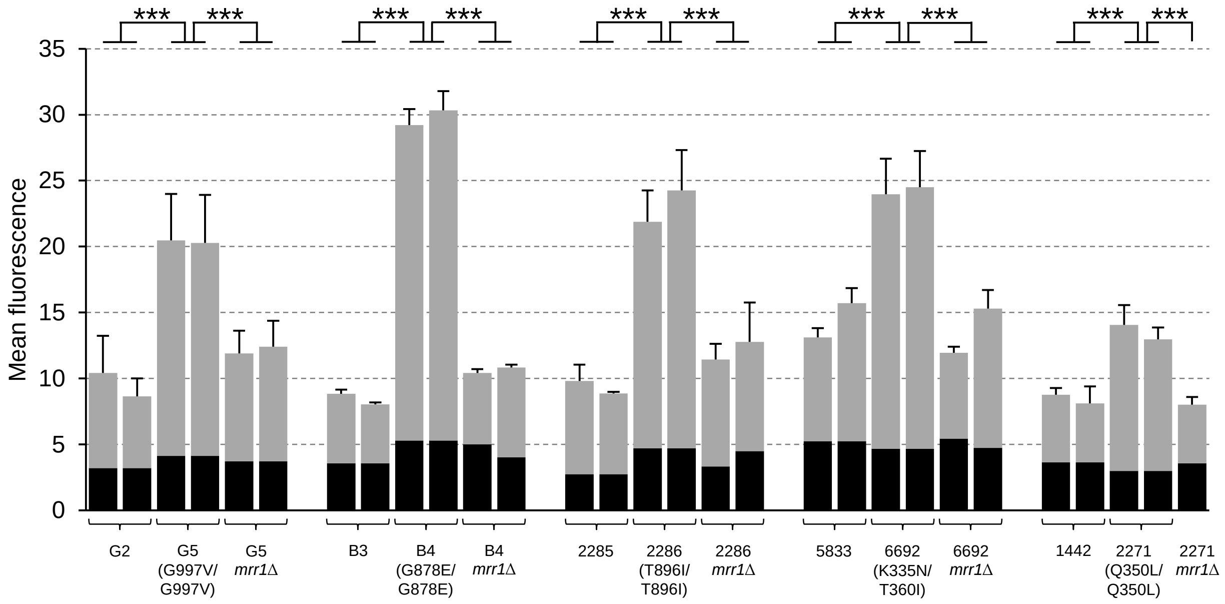

Figure S5 *FLU1* expression in the fluconazole-susceptible clinical isolates G2, B3, DSY2285 (2285), 5833, and 1442, the matched resistant isolates G5, B4, DSY2286 (2286), 6692, and 2271 containing the indicated *MRR1* GOF mutations, and two independent *mrr1*Δ mutants derived from each resistant isolate (only one *mrr1*Δ mutant for isolate 2271). A P_{FLU1}-*GFP* reporter fusion was integrated into the strains (two independent transformants of each clinical isolate were used) and the mean fluorescence of cells grown to log phase in YPD medium was determined by flow cytometry (means and standard deviations from at least three biological replicates). The background fluorescence of the parental strains without *GFP* is indicated by the black part of the columns. *GFP* reporter strains: G2FLU1G2A and -B (G2), G5FLU1G2A and -B (G5), G5MRR1M4FLU1G2A and -B (G5*mrr1*Δ), B3FLU1G2A and -B (B3), B4FLU1G2A and -B (B4), B4MRR1M4FLU1G2A and -B (B4*mrr1*Δ), DSY2285FLU1G2A and -B (2285), DSY2286FLU1G2A and -B (2286), DSY2286MRR1M4FLU1G2A and -B (2286*mrr1*Δ), 5833FLU1G2A and -B (5833), 6692FLU1G2A and -B (6692), 6692MRR1M4FLU1G2A and -B (6692*mrr1*Δ), 1442FLU1G2A and -B (1442), 2271FLU1G2A and -B (2271), 2271MRR1M4FLU1G2A (2271*mrr1*Δ). Control strains without *GFP*: G2, G5, G5MRR1M4A and -B (G5*mrr1*Δ), B3, B4, B4MRR1M4A and -B (B4*mrr1*Δ), DSY2285 (2285), DSY2286 (2286), DSY2286MRR1M4A and -B (2286*mrr1*Δ), 5833, 6692, 6692MRR1M4A and -B (6692*mrr1*Δ), 1442, 2271, 2271MRR1M4A (2271*mrr1*Δ). Significant differences (background-subtracted values, ANOVA) are marked with asterisks (*** $P < 0.001$).
